# Bio-Inspired Optimization in Soft Robot Design: Methods, Applications, and Future Directions

**DOI:** 10.3390/biomimetics11100714

**Published:** 2026-10-07

**Authors:** Yilun Sun, Tim C. Lueth

**Affiliations:** 1 Laboratory of Robotics and Multibody System, School of Mechanical Engineering, Tongji University, Shanghai 201804, China; 2 Institute of Micro Technology and Medical Device Technology, Technical University of Munich, 85748 Garching, Germany; tim.lueth@tum.de

**Keywords:** soft robotics, bio-inspired optimization, evolutionary algorithm, swarm intelligence, topology optimization, machine learning, additive manufacturing, design automation

## Abstract

Soft robots offer significant advantages in adaptability, safe interactions, and operation in unstructured environments, yet their design faces unique challenges due to infinite degrees of freedom, nonlinear material behavior, and coupled multi-physics interactions. Bio-inspired optimization methods have emerged as a powerful tool to navigate these complex design spaces. While individual optimization paradigms have been reviewed separately, no single survey has systematically compared all major method families side by side for soft robot design or provided cross-method selection guidelines. This review fills that gap with a broad, method-centric survey. We classify and compare evolutionary algorithms, swarm intelligence, topology optimization, and emerging machine-learning-assisted approaches. A cross-method comparison framework with practical selection guidelines is presented. Representative applications covering robot body design, actuator geometry, multi-material distribution, and sensing–control co-optimization are analyzed for each method category. Open-source simulation platforms and topology optimization codes are also catalogued. Finally, we outline future directions including computational scalability, optimization-to-fabrication integration, sim-to-real transfer, benchmarking and standardization, multi-objective optimization, and autonomous design pipelines from biological inspiration to fabricated prototypes.

## 1. Introduction

In nature, soft-bodied creatures such as octopuses, caterpillars, and jellyfish accomplish locomotion, manipulation, and environmental adaptation without relying on rigid skeletons [1,2,3,4]. These observations have inspired the field of soft robotics, which replaces the rigid links and joints of conventional robots with compliant structures made from elastomers, hydrogels, and other flexible materials [5,6]. Soft robots offer improved safety in human–robot interaction, superior conformability to irregular objects, and versatile whole-body locomotion enabled by a high number of degrees of freedom [5,7,8,9].

However, the design of soft robots presents fundamental challenges. Soft robots exhibit theoretically infinite degrees of freedom arising from continuous body deformation [10,11]. Their constituent materials are inherently nonlinear, viscoelastic, and often strain-rate-dependent [12,13]. Furthermore, soft robotic systems frequently involve coupled multi-physics interactions, such as fluid–structure coupling in pneumatic actuators, thermo-mechanical coupling in shape-memory alloy actuators, and electro-mechanical coupling in dielectric elastomer actuators [14,15,16,17]. These characteristics make closed-form analytical design methods often insufficient for realistic designs, and even finite element methods (FEM) become computationally expensive due to the geometric and material nonlinearities [18,19].

In recent decades, bio-inspired optimization methods have emerged as a powerful approach to navigate these complex design spaces [20,21]. Here, a broad interpretation is adopted for the term “bio-inspired optimization”, which encompasses all computational methods whose fundamental principles can be traced to biological optimization processes. This naturally includes evolutionary algorithms, which model Darwinian natural selection, and swarm intelligence, which replicates collective animal behavior. It also includes topology optimization, which originates from biological structural adaptation. Its variants such as growth-inspired shape optimization methods make this biological connection even more explicit. Machine learning (ML)-assisted methods also have bio-inspired roots of optimization, as artificial neural networks model biological neural systems, and reinforcement learning imitates trial-and-reward process through which animals progressively refine their behavior based on environmental feedback. Applying bio-inspired optimization to soft robot design is a natural fit, as the biological organisms that inspire these robots have been shaped by millions of years of evolutionary optimization [22]. In addition, these methods can handle high-dimensional, multi-modal, and non-convex search spaces without requiring gradient information, tolerate discontinuous objective functions, and naturally support multi-objective trade-offs [23,24]. These properties match the characteristics of soft robot design problems well, which involve vast design spaces spanning geometry, materials, actuation, and control, as well as competing goals such as maximizing displacement while minimizing actuation pressure.

This field has grown rapidly, driven by advances on multiple fronts: surrogate-assisted strategies, reinforcement-learning-based co-design, and generative models on the algorithmic side [25,26,27,28,29]; open-source simulation platforms such as SOFA, Voxelyze, and FEniCS [30,31,32]; and multi-material additive manufacturing that enables the physical realization of optimized designs with spatially varying stiffness [33,34]. Together, these developments are converging toward integrated design pipelines from biological inspiration through computational optimization to fabricated prototypes.

Several review articles have examined aspects of this landscape, but each addresses only a portion of the full picture, as illustrated in Table 1. Rus and Tolley [5] provided a foundational overview of soft robot design, fabrication, and control, but did not focus on optimization methods. Chen and Wang [35] offered an early review dedicated to design optimization of soft robots, covering topology optimization, shape optimization, and evolutionary approaches. However, that work did not cover more recent advances such as machine-learning-assisted optimization, reinforcement-learning-based co-design, and generative models. Pinskier and Howard [36] examined the transition from bioinspiration to computer-generated soft robot designs, covering evolutionary and generative approaches but not systematically addressing the full spectrum of optimization methods. Ahmed et al. [37] surveyed a decade of bio-inspired soft robots organized by actuation type, with limited attention to the computational design process. Hammond et al. [38] reviewed bioinspired soft robotics with emphasis on actuation, modeling, and control, but with limited attention to optimization. More recently, Stroppa et al. [39] presented an intensive survey of evolutionary and non-evolutionary computation methods for soft robot design and tracking, but did not cover machine-learning-assisted design approaches. Hasanshahi et al. [40] categorized soft robot design methods into bio-inspired, topology optimization, and evolutionary computation, but treated these in isolation without cross-method comparison or practical selection guidelines. Wang et al. [41] provided a broad multidisciplinary review of intelligent soft robotics, in which optimization is one aspect among many rather than the organizing principle.

The present review aims to fill these gaps by providing a unified, method-centric survey of bio-inspired optimization techniques for soft robot design. Our contribution is distinguished from existing reviews in the following aspects:

- **Broad methodological coverage:** We systematically classify and compare a broad range of bio-inspired optimization methods for soft robot design, spanning evolutionary algorithms, swarm intelligence, topology optimization, and machine-learning-assisted approaches.
- **Cross-method comparison with practical guidelines:** We present a comparative analysis across method categories, evaluating them against a variety of criteria. We provide decision guidelines to assist researchers and practitioners in selecting the most appropriate optimization approach for their specific design problem.
- **Simulation tools:** We catalogue open-source simulation platforms in the community, providing a practical resource for researchers entering the field.

The literature reviewed in this paper was identified through systematic searches of Web of Science, Scopus, and Google Scholar (last search performed on 1 September 2026). Keyword combinations such as “soft robot” AND “optimization”, “topology optimization” AND “soft actuator”, “evolutionary algorithm” AND “soft robot design”, “reinforcement learning” AND “soft robot morphology”, and “generative model” AND “robot design” were used for search. Additional relevant studies were identified through backward and forward citation tracking of key papers. Studies were included if they (i) presented or applied a computational optimization method to the design of a soft robotic system (e.g., actuator geometry, mechanism topology, morphology, or multi-material distribution), and (ii) reported design outcomes supported by simulation or experimental results. Publications focusing exclusively on soft robot control, sensing, or fabrication without a design optimization component were excluded from the main review but are cited where relevant for context. In total, 221 publications met the inclusion criteria and form the basis of this review.

The remainder of this paper is organized as follows. Section 2 provides background on soft robotics and the design challenges that motivate the use of optimization. Section 3 reviews bio-inspired optimization methods with a cross-method comparison. Section 4 surveys applications across major design domains and provides a taxonomy table. Section 5 discusses simulation tools. Section 6 identifies limitations, emerging trends, and future directions. Section 7 concludes the entire paper.

## 2. Background

### 2.1. Soft Robotics: Materials, Actuation, and Fabrication

#### 2.1.1. Definition and Distinguishing Features

Soft robots are mechanical systems constructed primarily from materials with elastic moduli on the order of those found in biological tissues (indicatively $10^{4}$–$10^{9}$ Pa), in contrast to the rigid metals and hard plastics (elastic moduli on the order of $10^{9}$–$10^{12}$ Pa) used in conventional robotics [6,42]. This material compliance allows soft robots to undergo large, continuous deformations rather than the discrete rotations of rigid-link mechanisms. As a result, soft robots can conform to irregular surfaces, distribute contact forces over large areas, and absorb impacts without damage. These properties can improve safety in human interaction and better adapt soft robots to unstructured environments [5,8,17].

A key distinguishing feature of soft robots is the coupling between structure, actuation, and sensing. In rigid robots, each joint is an independent actuated degree of freedom, and the overall system behavior is the sum of its parts. In soft robots, the body itself is both the structure and the mechanism, where deformation occurs throughout the entire body, and the morphology of the robot directly shapes its functional capabilities [43,44,45]. This tight coupling between form and function is a hallmark of biological organisms, which makes soft robots powerful but also challenging to design.

#### 2.1.2. Materials

The choice of material fundamentally determines the mechanical behavior, actuation range, and durability of a soft robot. The most commonly used materials include the following:

**Silicone elastomers:** Silicone-based materials, such as Ecoflex, Dragon Skin, and polydimethylsiloxane (PDMS), are the most widely used materials in soft robotics due to their high elongation at break, low Shore hardness, chemical inertness, and ease of processing through casting or 3D printing [46,47,48,49]. Their hyperelastic behavior is typically described by constitutive models such as the Mooney–Rivlin, Ogden, or Yeoh models [12].

**Thermoplastic elastomers and flexible polymers:** Thermoplastic polyurethane (TPU), thermoplastic elastomers (TPEs), and flexible polyamides (PAs) have gained increasing popularity in soft robotics, largely driven by their compatibility with different 3D printing technologies [50,51,52,53]. TPU offers a good combination of high elongation, abrasion resistance, and tunable Shore hardness, making it well suited for printing compliant actuators and grippers. TPEs provide rubber-like elasticity with the processability of thermoplastics, enabling rapid prototyping of soft robotic components. Flexible PA variants (e.g., PA 6, PA 12) offer higher strength and chemical resistance than TPU while retaining sufficient flexibility for semi-soft structures [53,54]. Compared to cast silicone elastomers, these thermoplastic materials enable faster design-fabrication-test iterations through 3D printing, which is particularly advantageous when optimization algorithms require physical validation of many candidate designs.

**Hydrogels:** Hydrogels are water-swollen polymer networks that can exhibit stimuli-responsive behavior, swelling or contracting in response to changes in temperature, pH, ionic concentration, or electric fields [55,56]. Their biocompatibility makes them attractive for biomedical soft robots, while their relatively low mechanical strength and slow response times limit their applicability in high-force or high-speed tasks.

**Shape memory polymers (SMPs):** SMPs can be programmed into a temporary shape and recover their original shape upon application of an appropriate stimulus, typically heat [57,58]. Multi-material 3D printing has enabled the fabrication of SMP-based soft robots with spatially programmed shape memory transitions, opening possibilities for four-dimensional (4D) printing of soft robotic structures [59].

**Dielectric elastomers:** Dielectric elastomer actuators (DEAs) consist of an elastomer membrane sandwiched between compliant electrodes. When a voltage is applied, electrostatic pressure compresses the membrane, causing it to expand in area [16,60,61]. DEAs can achieve high actuation strains, fast response times, and high energy densities, but require high driving voltages (typically in the kilovolt range).

#### 2.1.3. Actuation Principles

Soft robots employ a diverse range of actuation mechanisms, each with distinct advantages and limitations:

**Pneumatic and hydraulic actuation:** Pneumatic networks (PneuNets) are among the most widespread actuation strategies in soft robotics [14,62]. By selectively pressurizing internal chambers embedded within an elastomeric body, complex bending, twisting, and elongation motions can be generated. The geometry, number, and arrangement of these chambers largely determine the robot’s deformation behavior, making them a natural target for optimization. Hydraulic actuation follows similar principles but uses incompressible fluids, providing higher force output at the cost of increased system complexity [63].

**Cable-driven actuation:** Inspired by biological musculoskeletal systems, cable-driven soft robots use tendons routed through or along a compliant body to generate bending and contraction [64,65,66]. The routing path and attachment points of the cables directly influence the achievable motion, providing another rich design space for optimization.

**Electroactive actuation:** This category includes DEAs, ionic polymer–metal composites (IPMCs), and conducting polymer actuators, all of which convert electrical energy into mechanical deformation [16,67,68]. These actuators offer the advantage of direct electrical control but often face challenges in force output, efficiency, or operational lifetime.

**Thermally driven actuation:** SMPs actuate through temperature-induced phase changes or glass transitions [57]. Combustion-driven actuators represent a more extreme variant, using rapid gas expansion from controlled combustion to produce impulsive motions such as jumping [69].

**Magnetically driven actuation:** By embedding magnetic particles within an elastomeric matrix, soft robots can be actuated remotely through external magnetic fields [70]. The spatial distribution and orientation of the magnetic domains within the body govern the achievable deformation modes, making domain optimization a key design problem.

#### 2.1.4. Fabrication Methods

The fabrication method constrains what designs are physically realizable and therefore directly influences the design optimization process:

**Molding and casting:** The most established fabrication method for silicone-based soft robots involves pouring liquid elastomer into rigid molds, which are often produced by 3D printing, followed by curing and demolding [49,71,72]. Multi-step molding with sacrificial cores enables the creation of internal channels for pneumatic actuation. While reliable and accessible, molding is limited in geometric complexity and cannot easily produce multi-material structures or internal lattice architectures.

**Additive manufacturing (3D printing):** 3D printing has become a transformative enabler for soft robotics, allowing direct fabrication of complex geometries, internal channel networks, and multi-material structures that are impossible to achieve through conventional molding [33,73,74,75,76]. Key technologies include fused deposition modeling (FDM) with thermoplastic elastomers, direct ink writing (DIW) of silicone-based inks, stereolithography (SLA) of photopolymerizable elastomers, and multi-material PolyJet printing. The ability to deposit multiple materials with spatially varying stiffness in a single print is particularly important for optimization-driven design, as it enables the physical realization of computationally optimized material distributions [34,77,78].

**Lamination and layer-based fabrication:** Techniques such as shape deposition manufacturing and lamination of pre-fabricated layers offer an intermediate approach, enabling the integration of sensors, actuators, and structural elements within a layered soft robotic body [79,80].

### 2.2. Design Challenges to Soft Robots

#### 2.2.1. Infinite Degrees of Freedom

**Figure 1:**
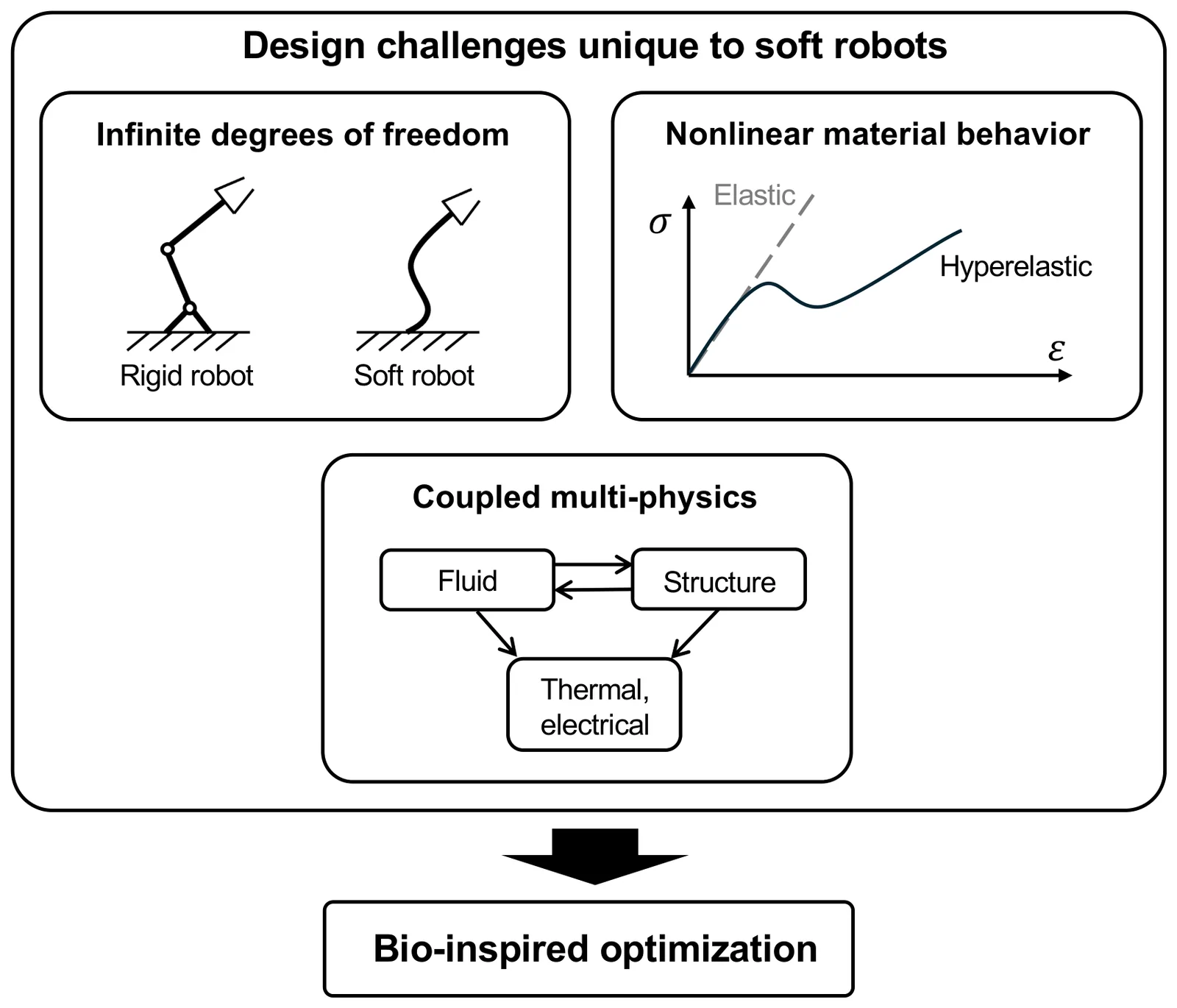
Design challenges to soft robots, which motivate the use of bio-inspired optimization methods.

Rigid robots are described by a finite number of joint coordinates, and their kinematics can be fully captured by established frameworks such as Denavit–Hartenberg parameterization. Soft robots, by contrast, are continuum structures whose configuration space is, in principle, infinite-dimensional [10,11] (see Figure 1). Any point on the body can undergo independent displacement, and the resulting deformation field is governed by partial differential equations rather than ordinary differential equations. This means that the design space is correspondingly vast, including not only the overall shape but also the local geometry, wall thickness, and internal architecture at every point. Traditional design-by-intuition approaches can explore only a small fraction of this space.

#### 2.2.2. Nonlinear Material Behavior

The materials used in soft robotics exhibit strongly nonlinear stress–strain relationships, large deformations, viscoelasticity, and, in some cases, anisotropy and rate dependence [12,13]. Constitutive modeling requires hyperelastic frameworks (e.g., Mooney–Rivlin, Ogden, Arruda–Boyce), and even these idealized models may not fully capture the material’s behavior under complex loading conditions. This nonlinearity means that the relationship between design parameters (e.g., chamber geometry) and performance metrics (e.g., tip displacement, grasping force) is highly nonlinear, often non-monotonic, and difficult to predict without simulation.

#### 2.2.3. Coupled Multi-Physics Interactions

Soft robotic systems typically involve the simultaneous interaction of multiple physical domains [30,81]: (a) **Fluid–structure interaction:** In pneumatically actuated soft robots, the pressurized air or fluid interacts with the deformable elastomeric structure, and the deformation of the structure in turn changes the fluid domain. (b) **Electro-mechanical coupling:** In DEAs, the electrostatic field depends on the deformed geometry of the membrane, which in turn depends on the electrostatic pressure and creates a strongly coupled, nonlinear problem. (c) **Thermo-mechanical coupling:** In SMP-actuated robots, the actuator force depends on the temperature distribution, which is influenced by the mechanical deformation and heat transfer conditions. These coupled interactions increase the computational cost of simulation and make the design problem inherently multi-disciplinary.

### 2.3. Why Bio-Inspired Optimization?

#### 2.3.1. Limitations of Traditional Optimization Approaches

Gradient-based optimization methods, such as steepest descent, conjugate gradient, and sequential quadratic programming, are efficient when the objective function is smooth, unimodal, and differentiable with respect to the design variables [82]. However, they face significant limitations in the context of soft robot design. First, gradient computation is often difficult or impossible when the objective is evaluated through a black-box FEM solver, particularly with contact, material failure, or topological changes. Second, gradient-based methods converge to the nearest local optimum and cannot escape basins of attraction to explore alternative designs. Third, they handle discrete or categorical variables (e.g., material type, number of chambers) poorly. While adjoint methods and differentiable simulation frameworks have made progress in providing gradients for some soft robot design problems [83,84], their applicability remains limited to specific problem formulations.

Trial-and-error design, guided by engineering intuition and biological inspiration, has produced many successful soft robots. However, it is inherently limited by the designer’s experience and imagination, and it cannot systematically explore the vast design spaces that characterize soft robotic systems. The result is that promising regions of the design space, particularly non-intuitive designs that a human designer would not consider, remain unexplored.

#### 2.3.2. Properties of Bio-Inspired Optimization

Bio-inspired optimization algorithms, as mentioned in Section 1, address the above limitations through several key properties inherited from their biological counterparts [23,24]:

**Population-based search:** Unlike gradient-based methods that track a single solution, bio-inspired algorithms maintain a population of candidate solutions that simultaneously explore different regions of the design space. This provides natural robustness against local optima and enables the discovery of diverse, high-performing designs.

**Gradient-free operation:** Some bio-inspired algorithms evaluate candidate solutions based on their fitness (objective function value) without requiring gradient information. This makes them compatible with any simulation tool, including black-box FEM solvers, and allows them to handle non-smooth, noisy, or discontinuous objective functions.

**Handling of mixed-variable spaces:** Many bio-inspired algorithms naturally accommodate continuous, discrete, and categorical design variables within the same optimization framework, making them well suited to soft robot design problems that involve both continuous geometric parameters and discrete material choices.

**Multi-objective capability:** Algorithms such as NSGA-II and MOEA/D are specifically designed to identify Pareto-optimal sets of solutions [85]. This is directly relevant to soft robot design, where trade-offs between performance metrics (e.g., displacement vs. stiffness, speed vs. energy consumption) are the norm.

**Scalability and parallelizability:** The population-based nature of bio-inspired algorithms makes them naturally suited to parallel evaluation, where multiple candidate designs can be simulated concurrently on multi-core processors or cloud computing infrastructure, partially offsetting the computational cost of FEM-in-the-loop optimization.

#### 2.3.3. Biological Analogy

There is a compelling conceptual symmetry in applying bio-inspired optimization to soft robot design. The biological organisms that serve as design inspiration for soft robots (e.g., octopuses, caterpillars, fish, plants) are themselves the products of evolutionary optimization [86,87]. They are shaped by natural selection over millions of years to achieve high performance within the constraints of soft, compliant materials [22]. Evolutionary algorithms and other bio-inspired methods replicate key aspects of this process in silico: variation (mutation and crossover), selection (fitness-based survival), and adaptation (population-level improvement over generations). From this perspective, bio-inspired optimization does not merely borrow algorithms from biology. It also mirrors the same process by which nature arrived at the soft-bodied designs that inspire the field.

Furthermore, the principles underlying different bio-inspired optimization methods (e.g., evolution, swarm behavior, ecological competition, growth and remodeling) capture different aspects of biological design processes. Evolutionary algorithms model the long-term adaptation of populations across generations. Swarm intelligence captures the collective decision-making of decentralized agents. Growth-inspired methods model the morphogenetic processes by which biological structures develop their shape. This diversity of biological principles provides researchers with a rich toolkit of algorithmic strategies, offering different strengths for different types of design problems.

## 3. Bio-Inspired Optimization Methods

**Figure 2:**
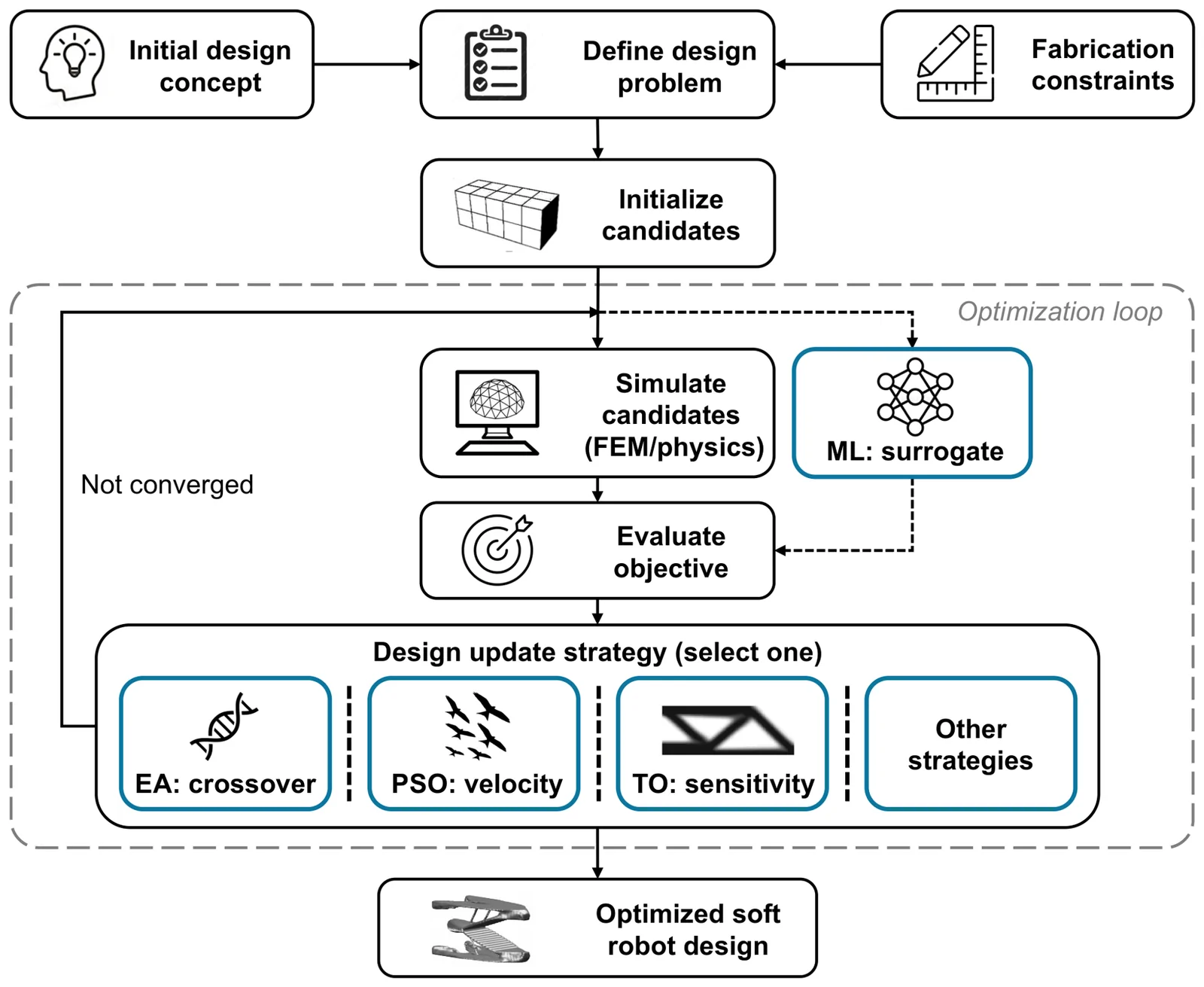
Generalized workflow of bio-inspired optimization for soft robot design. The optimization loop iterates between physics-based simulation (or an ML surrogate that approximates it) and a design update step. The design update block shows alternative optimization strategies reviewed in this article, and a given study typically employs one.

This section reviews the principal families of bio-inspired optimization methods that have been applied to soft robot design. As defined in Section 1, we interpret “bio-inspired optimization” broadly to include not only algorithms directly modeled on evolutionary and swarm behavior (Section 3.1, Section 3.2 and Section 3.3), but also topology optimization methods rooted in biological structural adaptation (Section 3.4), and machine-learning-assisted methods built on bio-inspired neural and learning architectures (Section 3.5). For each method category, we describe the underlying biological principles, the algorithmic mechanism, key variants, and representative work in soft robotics. Figure 2 illustrates the generalized optimization workflow shared by these methods. In each iteration, candidate designs are first evaluated through physics-based simulation or an ML surrogate. Their performance is then scored against one or more objectives, and a design update strategy generates improved candidates for the next iteration. These strategies are presented as selectable alternatives in Figure 2.

### 3.1. Evolutionary Algorithms

Evolutionary algorithms (EAs) are optimization methods inspired by the principles of biological evolution: variation, selection, and heredity [88,89]. A population of candidate solutions (individuals) is iteratively improved through stochastic operators that mimic natural selection and genetic recombination. EAs are among the most widely applied bio-inspired methods in soft robot design due to their flexibility, global search capability, and ability to handle complex, non-convex design spaces.

#### 3.1.1. Genetic Algorithms

Genetic algorithms (GAs) represent candidate designs as chromosome-like strings of variables (binary, integer, or real-valued) and evolve them through selection, crossover, and mutation operators [90,91]. In each generation, individuals are evaluated by a fitness function (typically computed via FEM simulation), and high-fitness individuals are preferentially selected to produce offspring. Crossover combines the design variables of two parent solutions, while mutation introduces random perturbations to maintain diversity and prevent premature convergence.

In soft robotics, GAs have been applied to optimize the geometry of pneumatic soft actuators [92,93], the cross-sectional topology of soft pneumatic chambers [94], the shape of soft grippers for maximum grasping force or adaptability [36], and the body plan of multi-legged soft locomotion robots [95,96]. An advantage of GAs is their straightforward integration with any simulation tool: the fitness function is treated as a black box, making GAs compatible with commercial FEM solvers (e.g., ABAQUS 2026 (Dassault, Vélizy-Villacoublay, France), ANSYS 2026 (Canonsburg, PA, USA)) as well as open-source platforms. However, GAs typically require a large number of fitness evaluations to converge, which can be difficult when each evaluation involves a computationally expensive FEM simulation.

#### 3.1.2. Evolutionary Strategies

Evolutionary strategies (ESs), including the covariance matrix adaptation evolution strategy (CMA-ES), operate primarily on continuous design variables and rely on self-adaptive mutation step sizes rather than crossover [97,98]. CMA-ES maintains a multivariate normal distribution over the design space and adaptively updates its covariance matrix to learn favorable search directions. This makes CMA-ES particularly effective for continuous parameter optimization problems with moderate dimensionality (typically up to a few hundred variables).

CMA-ES has been used to optimize the geometric parameters of soft pneumatic actuators, including chamber dimensions, wall thickness, and fiber reinforcement angles [39,99,100]. Its adaptive step-size control makes it well suited to the rugged, multi-modal landscapes typical of soft robot design, and it generally requires fewer function evaluations than standard GAs for continuous-variable problems.

#### 3.1.3. Genetic Programming and Compositional Pattern-Producing Networks

Genetic programming (GP) evolves programs or mathematical expressions rather than fixed-length parameter vectors, enabling it to discover novel design representations [101]. In soft robotics, GP has been extended through compositional pattern-producing networks (CPPNs), which encode spatial patterns of material distribution as functions of position [21,102]. A CPPN takes spatial coordinates as input and outputs local material properties (e.g., stiffness, presence/absence of material), allowing the representation of complex, high-resolution designs with a compact genotype. This encoding was central to the seminal work on evolving soft robots with multiple materials by Cheney et al. [21] and has been widely adopted in the evolutionary soft robotics community [25,103,104]. The CPPN-NEAT framework, which evolves both the topology and weights of CPPNs using the NEAT algorithm, provides an open-ended generative encoding that can produce designs of varying complexity [105].

### 3.2. Swarm Intelligence

Swarm intelligence algorithms draw inspiration from the collective behavior of decentralized, self-organized biological systems such as bird flocks, fish schools, ant colonies, and bee swarms [106,107,108]. Unlike evolutionary algorithms, which are inspired by inter-generational adaptation, swarm methods model the real-time cooperative search behavior of a population of agents.

#### 3.2.1. Particle Swarm Optimization

Particle swarm optimization (PSO) simulates the social behavior of bird flocking or fish schooling [24]. Each particle in the swarm represents a candidate design and moves through the design space influenced by its own best-known position and the best-known position of the entire swarm. The balance between individual memory (cognitive component) and social influence (social component) enables PSO to explore broadly while converging toward promising regions.

PSO has been applied to optimize structural parameters of soft bellows pneumatic actuators [109,110], to tune PID controller parameters for soft robot locomotion [111], and to calibrate simulation parameters for reducing the sim-to-real gap in soft robot models [112]. PSO is generally easier to implement and tune than GAs, with fewer hyperparameters, but may converge prematurely on highly multi-modal landscapes without appropriate diversity-preservation mechanisms.

#### 3.2.2. Ant Colony Optimization

Ant colony optimization (ACO) is inspired by the pheromone-based foraging behavior of ant colonies, where individual ants deposit chemical trails that collectively guide the colony toward optimal food sources [113]. ACO operates on discrete or combined design spaces and has been primarily applied in robotics for path planning and task allocation. In soft robot design, ACO has potential applicability to problems with combined structure, such as selecting optimal combinations of discrete actuator modules or routing internal channels within a soft body, while its application to soft robot morphology design remains limited compared to GAs and PSO [36].

### 3.3. Other Gradient-Free Metaheuristic Methods

Beyond evolutionary and swarm-based methods, several other gradient-free metaheuristics have also been applied to soft robot design.

#### 3.3.1. Simulated Annealing

Simulated annealing (SA) is inspired by the physical process of cooling a material from a high-temperature state to achieve a low-energy crystalline structure [114]. SA explores the design space by accepting not only improvements but also worsening moves, which enables the algorithm to escape local optima. SA has been applied to optimize material distributions and geometric parameters in compliant mechanism design [115]. Its single-solution nature makes it computationally lightweight but slower to converge than population-based methods.

#### 3.3.2. Differential Evolution

Differential evolution (DE) is a population-based metaheuristic that generates new candidate solutions by combining differences between existing population members [116]. DE is particularly effective for continuous optimization problems and has been applied to optimize the design parameters of soft actuators and compliant mechanisms [117,118]. Its simplicity, robustness, and competitive performance with minimal parameter tuning make it an attractive alternative to GAs for real-valued design optimization.

### 3.4. Topology Optimization

Topology optimization (TO) determines the optimal distribution of material within a given design domain to maximize certain performance subject to constraints [119,120]. Unlike the parametric optimization methods discussed above, which optimize a fixed set of design variables, TO can discover entirely new structural topologies. This makes TO particularly powerful for soft robot design, where the optimal internal architecture is unknown a priori.

#### 3.4.1. Density-Based Methods

The solid isotropic material with penalization (SIMP) method is the most widely used density-based TO approach [119,121]. Each finite element in the design domain is assigned a continuous density variable between 0 (void) and 1 (solid), and a penalization scheme drives intermediate densities toward discrete 0/1 values. The optimization is typically solved using gradient-based methods with sensitivities computed via adjoint analysis.

SIMP has been extensively applied to the design of soft pneumatic actuators and compliant mechanisms. Zhang et al. [122] used SIMP to optimize the three-dimensional shape of a soft gripper for maximum tip deflection under pneumatic pressure. Sun et al. [53] proposed LARG, a lightweight robotic gripper with 3D topology-optimized adaptive fingers that achieve compliant, adaptive grasping of irregular objects, with the optimized finger designs fabricated as single-piece 3D-printed structures. The same group further extended SIMP-based multi-objective optimization to the design of compliant robotic legs for bio-inspired quadruped robots, balancing structural stiffness and bending flexibility [54,123]. Caasenbrood et al. [124] adapted nonlinear SIMP-based TO with pressure-dependent loads for soft PneuNet design. A key challenge in applying SIMP to pneumatic soft robots is the design-dependent nature of the pressure load: as the structure deforms, the surface on which pressure acts changes, requiring specialized formulations. Kumar et al. [125,126] addressed this using Darcy’s law with a drainage term to model pressure load surfaces implicitly within the SIMP framework, and provided the open-source MATLAB code SoRoTop for topology optimization of pneumatic-driven soft robots [127]. More recently, multi-material SIMP formulations have been developed to simultaneously optimize the distribution of materials with different stiffnesses, enabling the design of soft robots with functionally graded properties [128,129].

#### 3.4.2. Level-Set and Phase-Field Approaches

Level-set methods represent the structural boundary as the zero level set of a higher-dimensional function, providing crisp material interfaces without the intermediate-density ambiguity of SIMP [130,131]. Phase-field approaches achieve a similar effect through a smooth but narrow transition zone governed by a diffusion-type equation. These methods have been applied to compliant mechanism design and are particularly advantageous when sharp interfaces between solid and void regions are important [132,133,134].

#### 3.4.3. Evolutionary Structural Optimization

Bi-directional evolutionary structural optimization (BESO) is a TO method that iteratively adds and removes material elements based on sensitivity information [135,136]. Unlike SIMP, BESO operates with discrete (0/1) density variables, producing designs that are inherently free of intermediate densities. Chen et al. [137] applied BESO to the topology optimization of soft pneumatic actuators with pressure-dependent loads, demonstrating an alternative approach to the design-dependent loading challenge. BESO bridges the gap between gradient-based TO and evolutionary algorithms: while it uses gradient information for sensitivity ranking, its update mechanism draws an analogy to evolutionary selection.

#### 3.4.4. Growth- and Remodeling-Inspired Methods

Growth-inspired TO methods draw directly from biological morphogenesis, the processes by which organisms develop their physical form [138,139,140]. Using these methods, material is added where mechanical stimuli indicate a need for reinforcement and removed where it is underutilized, analogous to Wolff’s law for bone remodeling. Growth-inspired approaches have been applied to compliant mechanism design and offer an intuitive interpretation of the optimization process [64]. They can be combined with density-based or level-set methods to guide the optimization toward biologically plausible topologies.

#### 3.4.5. Multi-Material and Multi-Physics Topology Optimization

Modern soft robot design increasingly requires simultaneous optimization of multiple materials with different mechanical properties. Multi-material TO extends standard SIMP or level-set formulations to optimally distribute two or more materials (e.g., a stiff polymer and a soft elastomer) within the design domain [27,128,129]. This is directly relevant to multi-material 3D printing, where spatially varying stiffness can be physically realized.

Multi-physics TO extends the optimization to coupled problems, such as simultaneous fluid–structure optimization of pneumatic channels [141] or electro-mechanical optimization of dielectric elastomer actuators [61]. These extensions significantly increase the computational cost but enable the design of soft robots that are optimized holistically rather than component by component.

### 3.5. Machine-Learning-Assisted Bio-Inspired Optimization

The computational cost of coupling bio-inspired optimization with high-fidelity FEM simulation has motivated the development of machine learning (ML)-assisted approaches. These methods use ML models to accelerate or replace parts of the optimization process.

#### 3.5.1. Surrogate-Assisted Evolutionary Optimization

Surrogate-assisted evolutionary optimization (SAEO) replaces part of the expensive FEM evaluations with predictions from a trained surrogate model, which is typically a Gaussian process, neural network, or radial basis function model [142,143]. The surrogate is trained on a small set of FEM simulations and then used to pre-screen candidate designs, directing the evolutionary search toward promising regions. Adaptive sampling strategies, such as expected improvement or lower confidence bound, guide when to query the true FEM simulation versus when to rely on the surrogate.

In soft robotics, surrogate-assisted approaches have been used to accelerate the optimization of pneumatic actuator geometries [144,145,146]. Yao et al. [147] proposed a generalized surrogate modeling pipeline based on reduced-order FEM models, validated through sim-to-real transfer across multiple soft actuator types. These approaches can reduce the number of FEM evaluations by one to two orders of magnitude, making optimization feasible for large-size problems.

#### 3.5.2. Bayesian Optimization

Bayesian optimization (BO) is a sequential, model-based optimization strategy that uses a probabilistic surrogate model and an acquisition function to balance exploration and exploitation [148,149]. BO is particularly well suited to expensive black-box optimization problems with low-to-moderate dimensionality.

BO has been applied to optimize the design parameters of soft pneumatic actuators [150], to tune morphological parameters of soft robots for specific tasks [151], and to perform multi-objective robot co-design through bilevel formulations [152]. Its sample efficiency makes it attractive when each design evaluation requires physical fabrication and testing rather than simulation alone. However, standard BO scales poorly beyond 20 design variables, limiting its applicability to parametric design problems [149].

#### 3.5.3. Reinforcement Learning for Morphology–Control Co-Optimization

Reinforcement learning (RL) offers a fundamentally different approach to soft robot design, which is to co-optimize the robot’s morphology and its control policy simultaneously, rather than optimizing a static design for a fixed objective [153,154]. The design is treated as part of the controller’s operating environment, and both are updated iteratively to maximize task performance (e.g., locomotion speed, manipulation accuracy).

This co-design paradigm has been explored extensively in simulated soft robot environments. Bhatia et al. [29] introduced Evolution Gym, a large-scale benchmark for evolving soft robots that co-optimizes voxel-based morphologies and neural network controllers. Kriegman et al. [25] demonstrated a scalable pipeline for designing reconfigurable organisms. In that work, an evolutionary algorithm was used to optimize the morphology and controller to orchestrate the behavior of the cells. More recently, approaches combining RL with differentiable simulation have enabled gradient-based co-optimization, achieving faster convergence than purely evolutionary methods [83,155]. Strgar and Kriegman [156] proposed morphological pretraining, where a universal morphology-agnostic controller is obtained through differentiable simulation and enables rapid evaluation of evolutionary design changes.

A key challenge in RL-based co-design is the sim-to-real gap, as controllers and morphologies optimized in simulation may not transfer directly to physical hardware due to modeling inaccuracies. To bridge this gap, surrogate compliance models capturing essential mechanical effects within a rigid-body simulator have been proposed recently [157].

#### 3.5.4. Generative Models for Soft Robot Design

Generative models, including variational autoencoders (VAEs), generative adversarial networks (GANs), and diffusion models, represent the latest advances in ML-assisted soft robot design [158,159,160]. These models learn the underlying distribution of viable designs from a dataset and can generate novel designs that lie within or beyond the training distribution.

Wang et al. [26] introduced DiffuseBot, a physics-augmented diffusion model that generates soft robot morphologies optimized for diverse tasks. By augmenting the diffusion process with a differentiable physics simulation, DiffuseBot can generate plausible designs. Hu et al. [161] applied GANs to modular robot design optimization, learning a latent design space from which new morphologies can be sampled and evaluated.

Compared to traditional evolutionary search, generative models can leverage prior knowledge encoded in training data, generate diverse designs in a single forward pass, and potentially discover design families rather than individual optima. However, they require large training datasets of evaluated designs, which may be expensive to generate. In addition, ensuring the physical validity and fabricability of generated designs remains an open challenge.

### 3.6. Comparison and Selection Criteria

The methods reviewed above differ substantially in their operational characteristics. Hence, selecting an appropriate method for a given soft robot design problem requires balancing multiple considerations. Table 2 provides a comparative overview across key criteria. The following discussion interprets these characteristics in terms of practical trade-offs for soft robot design. Here, the qualitative ratings and quantitative ranges in the tables are indicative estimates synthesized from the reviewed literature rather than results of controlled benchmarking. They are intended as starting points for method selection and should be validated against the specific problem at hand.

Evolutionary algorithms (GAs, CMA-ES) offer the broadest applicability, as they impose no requirements on the objective function and can interface with any simulation tool as a black box [88,89]. However, they typically require 10^2^–10^4^ fitness evaluations to converge [92,93,96], which becomes prohibitive when each evaluation involves a nonlinear FEM simulation. CMA-ES is more sample-efficient than standard GAs for continuous problems [97,98] but does not handle discrete or mixed variables. The CPPN-NEAT encoding enables open-ended design exploration but can produce geometrically complex structures that are difficult to fabricate [21,105]. In general, evolutionary methods are best suited to problems where the design space is poorly understood and broad exploration is more valuable than rapid convergence.

Swarm intelligence methods (PSO, ACO) have seen comparatively limited adoption in soft robot design. PSO has been applied mainly to low-dimensional parametric tuning [109,110,111,112] rather than topology discovery, and ACO’s discrete nature restricts it to combinatorial problems such as channel routing [113]. Systematic benchmarking of swarm methods against EAs on standardized soft robot problems remains scarce, making it difficult to assess when they offer genuine advantages over GAs or CMA-ES.

Simulated annealing (SA) and differential evolution (DE) provide lightweight alternatives. SA is computationally inexpensive but converges slowly on high-dimensional problems due to its single-solution search [114,115]. DE offers competitive performance with minimal hyperparameter tuning and is particularly effective for continuous parametric optimization [116,117,118]. A broader concern in this category is that SA’s single-solution search restricts its scalability and multi-objective capability, while DE’s restriction to continuous variables limits its applicability to problems with discrete or mixed design representations.

Topology optimization (SIMP, level-set, BESO) is the most powerful approach for structural discovery, as it operates at per-element resolution and can find non-intuitive topologies that parametric methods cannot reach [119,120]. Its gradient-based nature makes it highly efficient, typically converging in $10^{1}$–$10^{2}$ iterations [121,127]. However, TO faces specific challenges in soft robotics: large-deformation formulations are prone to convergence difficulties [124,137], design-dependent pressure loads require specialized formulations such as Darcy-based approaches [125,126], and optimized designs often contain features that violate manufacturing constraints such as thin walls or enclosed voids [163,164]. Multi-material TO expands the design space significantly but increases computational cost and regularization complexity [128,129].

Machine-learning-assisted methods address the evaluation cost bottleneck but introduce their own limitations. Surrogate models can reduce FEM evaluations by one to two orders of magnitude [144,145,146,147] but may produce inaccurate predictions in unexplored regions of the design space. Bayesian optimization is highly sample-efficient but scales poorly beyond approximately 20 design variables [148,149]. RL-based co-design produces impressive results in simulation [29,156], yet sim-to-real transfer remains a persistent challenge with few demonstrated physical deployments [157]. Generative models can propose novel designs in a single forward pass [26,161] but require large training datasets and offer limited guarantees on physical validity. Across all ML-assisted methods, uncertainty quantification in design predictions remains an open gap.

A recurring observation across all method families is the gap between simulation-based optimization and experimental validation. The majority of studies in the surveyed literature present results exclusively in simulation. Studies that demonstrate complete pipelines from optimization through fabrication to physical testing [53,54,124,127] remain the minority but provide stronger evidence of physical realizability. This gap is particularly pronounced in locomotion morphology design and RL-based co-design, while gripper and actuator design show greater maturity in closing the optimization-to-fabrication loop [53,122,165]. Based on the comparison above and the literature reviewed, we offer the following practical guidelines for method selection:

- **Parametric optimization of a soft actuator with known topology** (e.g., optimizing chamber dimensions, wall thickness, fiber angle): CMA-ES or Bayesian optimization are recommended for their sample efficiency in continuous, moderate-dimensional spaces. For problems with mixed or discrete variables, GAs or DE are preferable.
- **Topology discovery for a certain soft robotic structure** (e.g., finding the optimal internal structure of a soft actuator from scratch): SIMP-based or level-set TO is the recommended method, particularly when adjoint sensitivities can be computed. For problems where gradient computation is difficult, BESO or GA-based topology search can serve as alternatives.
- **Multi-material design** (e.g., distributing stiff and soft materials within a printable design domain): Multi-material SIMP or multi-material level-set TO is most appropriate, with GA-based search as a gradient-free alternative for complex multi-physics problems.
- **Morphology–control co-design** (e.g., simultaneously designing a soft robot’s body and locomotion controller): RL-based co-optimization or nested evolutionary approaches are recommended. Evolution Gym and similar benchmarks provide standardized testing environments.
- **Expensive evaluation with limited budget** (e.g., each design requires physical fabrication and testing): Bayesian optimization or surrogate-assisted EA approaches are recommended to minimize the number of evaluations.
- **Exploring novel, non-intuitive designs** (e.g., discovering fundamentally new soft robot morphologies): CPPN-NEAT, generative models, or large-scale evolutionary searches in voxel-based simulation environments are appropriate.
- **Multi-objective optimization** (e.g., balancing displacement, stiffness, weight, and fabricability): NSGA-II, MOEA/D, or multi-objective TO are recommended choices. Multi-objective BO can be used when evaluations are very expensive.

## 4. Applications in Soft Robot Design

**Figure 3:**
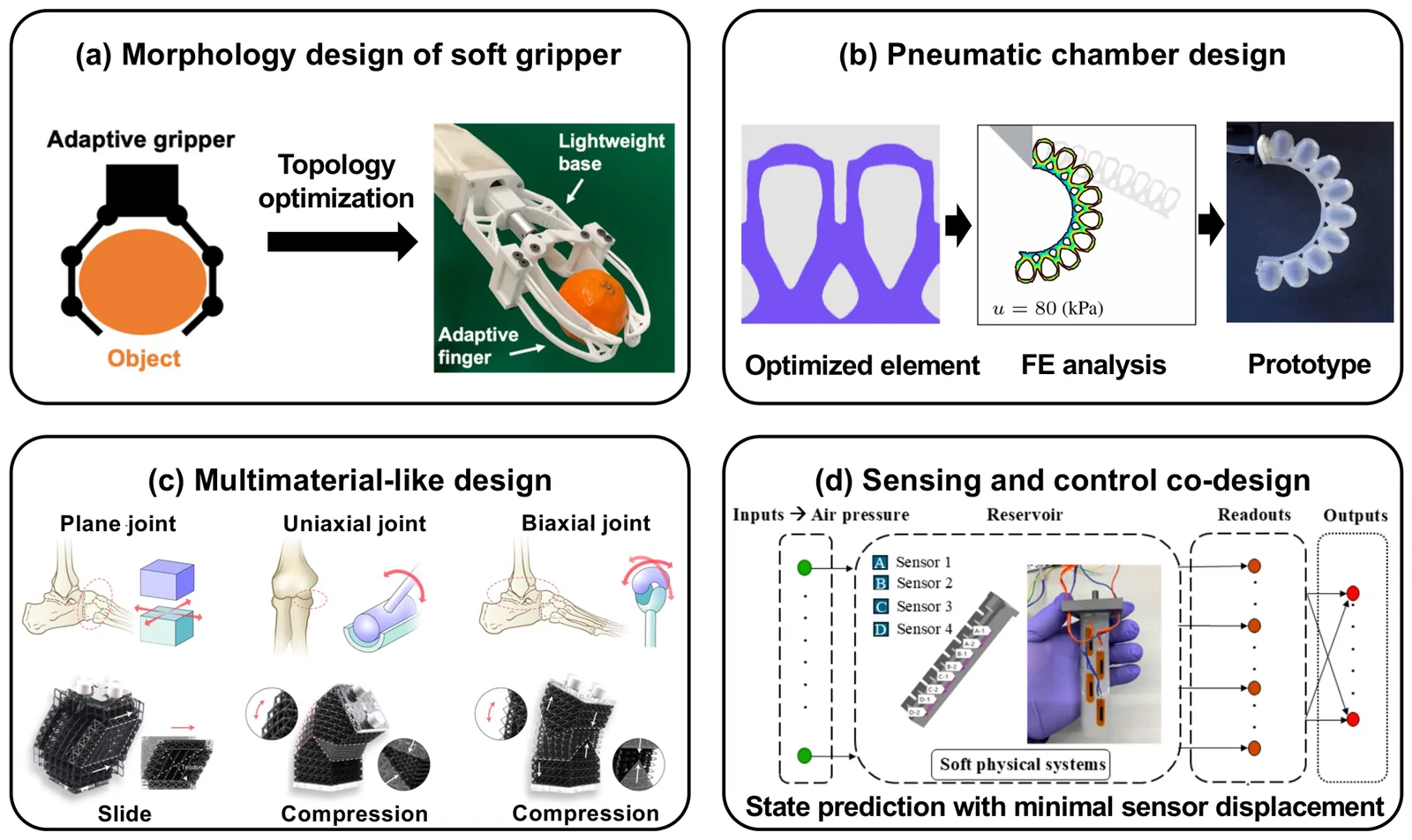
Representative applications of bio-inspired optimization in soft robot design. (**a**) Morphology and body design: topology optimization generates an adaptive finger structure from a design domain, yielding a lightweight, 3D-printed gripper (adapted from [53]). (**b**) Actuator and mechanism design: topology-optimized internal structure of a soft pneumatic actuator (adapted from [166]). (**c**) Multi-material design: bio-inspired compliant joint types mimicking human skeletal joints, fabricated using programmable 3D-printed microstructures (adapted from [167]). (**d**) Sensing and control co-design: optimal sensor placement within a soft pneumatic system (adapted from [168]).

The optimization methods reviewed in Section 3 have been applied to a wide range of soft robot design problems. This section surveys these applications organized by design domain, with representative examples illustrated in Figure 3.

### 4.1. Morphology and Body Design

#### 4.1.1. Locomotion Bodies

The design of soft robots for locomotion has been a primary testbed for bio-inspired optimization. Locomotion is particularly well suited to evolutionary optimization because the performance metric (e.g., speed, distance, energy efficiency) is straightforward to evaluate in simulation. **Voxel-based evolutionary design:** A widely adopted approach models soft robots as assemblies of discrete voxels, each assigned a material type (e.g., soft, rigid, actuated, passive), and evolves the arrangement using evolutionary algorithms. Cheney et al. [21] pioneered this approach using CPPN-NEAT to evolve soft robots with multiple materials in the Voxelyze simulator, producing diverse locomotion morphologies including crawling, undulating, and galloping gaits. Kriegman et al. [25] extended this pipeline to design reconfigurable biological robots (xenobots) from frog cells, demonstrating that computationally evolved morphologies can be physically realized in biological substrates. Bhatia et al. [29] introduced Evolution Gym, a large-scale benchmark environment for co-designing voxel-based soft robot morphologies and controllers, enabling standardized comparison across algorithms. More recently, Medvet et al. [162] investigated biodiversity in evolved voxel-based soft robots, showing that the choice of evolutionary algorithm and design representation significantly influences the diversity of evolved morphologies.

**Topology-optimized locomotion:** Kobayashi et al. [27] demonstrated that density-based topology optimization, combined with the material point method (MPM) for simulating large-deformation dynamics, can synthesize locomotive soft robots from scratch. Their method automatically discovered walking and climbing body plans without prescribing the locomotion mode, representing a milestone in the application of TO to soft robot locomotion.

**Bio-inspired morphologies:** Many locomotion designs draw directly from specific biological organisms. Caterpillar-inspired crawling robots have been optimized for wave-like peristaltic locomotion through parameter optimization of segment geometry and actuation timing [169]. Fish-inspired swimming robots have been designed using shape optimization and differentiable simulation to match desired hydrodynamic performance [170]. Ma et al. [171] proposed DiffAqua, a differentiable computational design pipeline for soft underwater swimmers that uses shape interpolation to explore morphological variations. Octopus-inspired manipulators and swimmers have motivated the optimization of continuum arm geometries and multi-arm coordination strategies [172].

#### 4.1.2. Grippers and Manipulators

Soft grippers represent one of the most commercially relevant applications of soft robot design optimization. The design challenge is to produce a gripper that can conform to objects of varying shapes and sizes while generating sufficient grasping force.

Zhang et al. [122] performed topology optimization of a 3D-printed soft gripper using SIMP, optimizing the outer shell topology for maximum tip deflection under pneumatic pressure. Zhang et al. [128] extended this to multi-material topology optimization of soft fingers for grippers, rehabilitation devices, and artificial hands. Liu et al. [173] used a GA-based approach to optimize the design of a soft robotic gripper for grasping unknown objects, balancing grasp adaptability and force output. Sun et al. [165] developed a 3D topology optimization framework for soft robotic grippers and proposed LARG, a lightweight gripper with topology-optimized adaptive fingers [53]. Pinskier et al. [174] combined multi-material topology optimization with multi-dimensional archive of phenotypic elites (MAP-Elites) quality-diversity search to generate diverse gripper designs that span a range of grasping behaviors, and further explored diversity-based topology optimization of soft robotic grippers [175]. More recently, Xie et al. [176] combined topology optimization with MAP-Elites to traverse complex design spaces, identifying high-performing designs across diverse behavioral niches.

### 4.2. Actuator and Mechanism Design

While morphology design addresses the overall body plan, actuator and mechanism design focuses on the internal functional components that generate motion: pneumatic chambers, cable routing paths, and compliant joint elements.

#### 4.2.1. Pneumatic Network (PneuNet) Optimization

Pneumatic soft actuators generate motion through the inflation of internal chambers embedded within an elastomeric body. The geometry, number, spacing, and shape of these chambers critically determine the actuator’s deformation behavior, making them natural targets for optimization.

Mosadegh et al. [14] established the foundational PneuNet architecture with rapidly actuating pneumatic networks. Subsequent optimization studies have used GAs to optimize chamber dimensions for maximum bending curvature [92], Bayesian optimization to tune bellow actuator geometry [150], and SIMP-based topology optimization to discover optimal chamber topologies from scratch [124,125]. Caasenbrood et al. [124] developed a computational design framework for pressure-driven soft robots through nonlinear topology optimization, addressing the challenge of design-dependent pressure loads. Kumar [127] provided the open-source SoRoTop MATLAB code for topology optimization of pneumatic-driven soft robots, lowering the barrier to entry for researchers. Mosser et al. [94] combined Convolutional Neural Network (CNN)-based surrogate models with Bézier curve-parameterized GAs to optimize the inner cavity structure of 3D soft pneumatic actuators. Dalklint et al. [141] proposed simultaneous shape and topology optimization of inflatable soft robots, treating both the external shape and internal material distribution as design variables. This unified approach avoids the sequential design paradigm (first optimize topology, then optimize shape) and can discover designs that are globally superior.

#### 4.2.2. Compliant Joint Synthesis

Compliant joints, also known as flexure hinges, replace conventional pin or ball joints with geometrically tailored material sections that undergo elastic deformation. In soft robotics, compliant joints serve as the fundamental building blocks for monolithic, 3D-printable robotic structures, eliminating the need for assembly of discrete parts [177,178,179]. The geometry and topology of a compliant joint directly determine its rotational range, stiffness, stress distribution, and fatigue life, making it a natural target for optimization.

Topology optimization has been the dominant method for compliant joint design since the foundational work of Sigmund [180], who formulated the problem as maximizing a displacement objective subject to stiffness and volume constraints. Mutlu et al. [181] investigated various flexure hinge morphologies for 3D-printed soft monolithic prosthetic fingers, identifying optimal hinge types that maximize bending displacement under minimal actuation force. Hussain et al. [182] demonstrated how to 3D-print compliant joints with a selected stiffness for cooperative underactuated soft grippers. More recently, topology-optimized compliant joints have been extended to continuum robots, where multi-axis flexure joints with optimized torsional stiffness and rotational flexibility enable compact, 3D-printable continuum structures [183,184]. In addition, a fish-tail-inspired shape optimization method was developed in [64] to improve the stress distribution across serially connected compliant joints in tendon-driven continuum robots.

### 4.3. Multi-Material Design

One of the most significant opportunities enabled by modern additive manufacturing is the spatial distribution of multiple materials with different mechanical properties within a single soft robot. Optimization methods are essential for exploiting this expanded design space, as the number of possible material configurations grows exponentially with the resolution of the print.

#### 4.3.1. Spatially Varying Stiffness

By varying the local stiffness across a soft robot’s body, designers can program its deformation behavior without altering the external geometry [167]. Stiff regions act as structural elements that resist deformation, while soft regions act as compliant joints or actuation zones. Multi-material topology optimization determines where to place each material to achieve the desired functional behavior.

Zhang et al. [128] demonstrated multi-material topology optimization of soft pneumatic fingers using a combination of hard and soft photopolymers, fabricated on a multi-material PolyJet printer. The optimized multi-material design significantly outperformed single-material designs in both deformation range and payload capacity. Sun et al. [52] applied optimization-based shape design to soft–rigid hybrid fingers, integrating topology-optimized compliant structures with multi-material fabrication for adaptive grasping. Schaffner et al. [34] used 3D printing of robotic soft actuators with programmable bioinspired architectures, where the spatial arrangement of stiff and soft regions was inspired by biological muscle-tendon systems.

#### 4.3.2. Functionally Graded Soft Structures

Functionally graded materials (FGMs) extend the concept of spatially varying stiffness from discrete multi-material distributions to continuous gradients of material properties. Grayscale digital light processing (DLP) 3D printing enables the fabrication of FGMs by locally varying the crosslinking density of a photopolymer, producing continuous stiffness gradients within a single print [185]. Evolutionary algorithms and TO methods have been applied to optimize these gradients for soft actuators, where a smooth stiffness transition can reduce stress concentrations and improve fatigue life compared to abrupt material interfaces [186].

#### 4.3.3. Heterogeneous Material Optimization for Additive Manufacturing

A key challenge in multi-material optimization is ensuring that the optimized design is compatible with the constraints of the fabrication process. Multi-material FDM printing imposes minimum feature sizes, limits on the number of simultaneously printable materials, and interface bonding constraints. PolyJet printing offers higher material resolution but is limited to specific photopolymer families. Recent work has begun to incorporate manufacturing constraints directly into the topology optimization formulation, using filtering and projection techniques to enforce minimum feature sizes, overhang constraints, and material interface requirements [163,164]. This integration of fabrication awareness into the optimization loop is essential for closing the gap between computationally optimized and physically realizable designs.

### 4.4. Sensing and Control Co-Design

The integration of sensing and control into the design optimization process represents an emerging frontier in soft robotics [187]. Rather than designing the mechanical structure first and then adding sensors and controllers post hoc, co-design approaches optimize all aspects simultaneously. Spielberg et al. [84] introduced a neural architecture that learns a salient and sparse selection of sensor placements for optimal task performance, evaluating their approach on six soft robot morphologies for tactile sensing and proprioception tasks. Truby et al. [188] demonstrated distributed proprioception of 3D configuration in soft, sensorized robots using deep learning, where the sensor layout was designed to provide sufficient observability of the robot’s shape. More recently, theoretical frameworks have been proposed to guide sensor design and placement by addressing how many sensors are sufficient for accurate state prediction in compliant systems [168].

### 4.5. Summary of Applications

Table 3 provides a taxonomy mapping application domains to optimization methods, biological inspirations, and representative references. This table is intended as a practical reference for researchers to identify which optimization methods have been applied to their domain of interest.

## 5. Simulation Tools

Simulation is the backbone of optimization-driven soft robot design, as each candidate design must be evaluated before the optimizer can update the population or design variables. The choice of simulation platform directly affects the fidelity, speed, and scope of the optimization. Table 4 summarizes the major platforms and their characteristics.

### 5.1. Finite Element Method Platforms

**SOFA (Simulation Open Framework Architecture):** SOFA is a widely used open-source framework for real-time simulation of deformable bodies, originally developed for medical simulation and subsequently extended to soft robotics [30,198]. The SoftRobots plugin provides dedicated components for pneumatic actuation, cable-driven actuation, and inverse modeling capabilities for control [199,200]. SOFA’s real-time performance makes it suitable for optimization loops that require moderate fidelity, though its linear FEM formulations may lack accuracy for the extreme nonlinearities encountered in some soft robot designs. Recent work has extended SOFA with interfaces for magnetic soft robot simulation [201].

**FEniCS:** FEniCS is an open-source computing platform for solving partial differential equations [32,202]. It provides a high-level Python interface and supports nonlinear hyperelastic formulations, making it suitable for high-fidelity simulation of soft robot mechanics [203]. FEniCS has been used for topology optimization of compliant mechanisms and soft actuators, particularly in conjunction with adjoint-based sensitivity analysis [204]. Compared to SOFA, the flexibility of FEniCS comes at the cost of requiring more user expertise to set up soft robot-specific simulations.

**Sorotoki:** Sorotoki is an open-source MATLAB toolkit that integrates design, modeling, and control of soft robots within a single framework [166]. It combines implicit CAD modeling via signed distance functions, nonlinear FEM simulation with hyperelastic materials, reduced-order Cosserat beam models, SIMP-based topology optimization with Method of Moving Asymptotes (MMA) solver, real-time fluidic control, and vision-based sensing. Four open-hardware 3D-printable soft robotic systems are also provided, making it an accessible entry point for researchers seeking an integrated design-to-control workflow.

**Commercial FEM solvers (ABAQUS, ANSYS, COMSOL 6.4):** Commercial solvers offer mature, well-validated nonlinear FEM capabilities and are widely used in the soft robotics community for design validation. They support hyperelastic material models, large deformations, fluid–structure interaction, and contact mechanics. However, their closed-source nature, licensing costs, and limited scripting flexibility can make the integration of optimization loops challenging. Optimization studies using commercial solvers typically rely on batch scripting, including writing input files, running the solver externally, and parsing output files, which inevitably adds implementation complexity [133,192,205].

### 5.2. Voxel-Based Simulators

**Voxelyze and Evosoro:** Voxelyze is an open-source voxel-based physics engine that models soft multimaterial objects as lattices of interconnected voxels with mass-spring-damper dynamics [31]. Evosoro provides a high-level Python wrapper around Voxelyze, enabling rapid setup of evolutionary optimization experiments for voxel-based soft robots [206]. Voxelyze has become the de facto standard for evolutionary soft robot design, used in seminal studies on evolving soft locomotion [21,25] and widely adopted as a benchmark simulator. A GPU-accelerated reimplementation, voxcraft-sim, has been developed to address computational bottlenecks for large-scale evolutionary searches [193].

**Evolution Gym:** Evolution Gym is a 2D voxel-based soft robot simulation environment, which is specifically designed as a co-design benchmark [29]. It provides a suite of standardized tasks (walking, climbing, catching, manipulating) with consistent evaluation protocols, as well as a user-friendly Python (v3.14.8) interface compatible with standard RL frameworks. Evolution Gym has become the primary benchmark for comparing morphology-control co-design algorithms and has catalyzed significant progress in the field [207,208].

### 5.3. Differentiable Simulators

Differentiable simulators compute gradients of the simulation output with respect to design and control parameters, enabling gradient-based optimization of soft robot morphology and control. ChainQueen [83] was an early differentiable simulator for soft robotics based on the material point method (MPM). DiffTaichi [194] provides a framework for building differentiable physical simulations using the Taichi programming language, supporting soft body, fluid, and rigid body dynamics. DiffRedMax [209] extends differentiable simulation to articulated and soft–rigid hybrid systems. These tools have enabled differentiable co-design [83,155] and physics-augmented generative models [26]. They represent a growing trend toward gradient-informed optimization that complements gradient-free bio-inspired methods.

### 5.4. General-Purpose Physics Engines

General-purpose physics engines such as MuJoCo [195], PyBullet [196], and Isaac Gym [197] have increasingly incorporated soft body simulation capabilities. While historically focused on rigid-body robotics, their GPU acceleration, RL integration, and large user communities make them also attractive for soft robot co-design research.

### 5.5. Topology Optimization Codes

Open-source topology optimization codes play a particularly important role in lowering the barrier for researchers to apply TO to soft robot design. Sigmund’s foundational 99-line MATLAB code [121] for 2D topology optimization has grown to a large family of educational codes [210,211,212,213]. For soft robotics specifically, Kumar [127] developed SoRoTop, a MATLAB code for topology optimization of pneumatic-driven soft robots with design-dependent pressure loads. Other researchers [214,215] developed MATLAB-based multi-material topology optimization frameworks using SIMP with MMA/OC solvers. These codes provide an accessible starting point for researchers seeking to apply density-based TO to soft robot design problems. The TopOpt group at DTU has released a series of web-based and downloadable topology optimization tools that can be adapted for compliant mechanism and soft actuator design [216]. While not specifically targeted at soft robotics, these tools provide validated implementations of standard SIMP, BESO, and level-set methods.

## 6. Discussion: Challenges and Future Directions

The preceding sections have surveyed methods, applications, and tools for bio-inspired optimization in soft robot design. While substantial progress has been made, several fundamental challenges remain. This section discusses each challenge in terms of what has been achieved, what gaps persist, and what research directions are most promising:

**Computational Cost and Scalability:** Surrogate-assisted optimization [144,145,146,147], GPU-accelerated simulators [193,194], and differentiable simulation [83,155] have significantly reduced the computational burden of optimization-driven soft robot design. However, scaling to three-dimensional, multi-physics, multi-material problems with fine spatial resolution remains difficult, as most studies are still limited to 2D or coarse 3D formulations [217]. GPU-accelerated simulators currently support mass-spring or MPM physics but not the nonlinear hyperelastic FEM that many soft robot applications require. Promising directions include GPU-native nonlinear FEM solvers, neural operator surrogates that generalize across geometries, and multi-fidelity strategies that combine coarse and fine simulations within a single optimization loop.

**Optimization-to-Fabrication Integration:** Manufacturing constraint filters such as minimum feature size and overhang penalties have been incorporated into TO formulations [163,164], and several studies have demonstrated complete optimization-to-fabrication-to-testing pipelines for soft grippers [53,165], robot legs [54,123], and pneumatic actuators [124,127]. Nevertheless, these constraints are typically process-specific and do not generalize across fabrication technologies. Enclosed internal voids, multi-material interface bonding, and support removal challenges are rarely addressed during optimization. Practical next steps include developing standardized fabrication constraint libraries as plug-in modules for TO solvers. Closed-loop workflows that feed measured print deviations back into the optimization would further help bridge the gap between computational and physical designs.

**Material Modeling and Sim-to-Real Transfer:** Hyperelastic constitutive models are now standard in soft robot simulation toolkits [30,166]. Strategies such as domain randomization and surrogate compliance models [157] have improved sim-to-real transfer for specific cases. However, standard models fail to capture viscoelasticity, hysteresis, stress softening, and material aging [218], all of which significantly affect real-world performance under cyclic loading. Batch-to-batch material variability and printing-induced anisotropy further widen this gap. Promising paths forward include probabilistic models that capture manufacturing variability, data-driven constitutive models trained on experimental data, and digital twin frameworks [219] that calibrate simulation against physical sensor data in real time.

**Benchmarks and Standardization:** Evolution Gym [29] provides a standardized benchmark for voxel-based co-design, and SoRoTop [127] defines benchmark problems for pneumatic soft robot TO. Beyond these, the field lacks widely adopted benchmarks for soft gripper design, multi-material distribution, compliant joint synthesis, or sensor placement optimization. Without shared benchmarks, cross-study comparison remains unreliable, and it is difficult to assess whether newly proposed methods represent genuine progress. A coordinated benchmark initiative covering gripper design, multi-material optimization, and compliant joints would fill this gap. Such an initiative should define standardized design domains, evaluation metrics, and open-source reference implementations.

**Multi-Objective Optimization:** Multi-objective methods such as NSGA-II and MOEA/D have been applied to soft robot design involving two or three competing objectives [54,85,123,220]. However, real-world design involves many more simultaneous considerations, including performance, weight, energy consumption, fabrication cost, durability, and safety. Many-objective optimization (four or more objectives) and the integration of sustainability metrics such as material usage and recyclability remain largely unexplored. Many-objective algorithms combined with interactive visualization tools and human-in-the-loop preference injection [221] could make such optimization practical for industrial soft robot design.

**Toward Autonomous Design Pipelines:** The trends discussed above are converging toward autonomous pipelines spanning task specification, optimization, fabrication-aware refinement, 3D printing, and experimental validation with feedback. Elements of this pipeline exist individually [25,53,127,147], but no existing system integrates them in a fully automated manner. A realistic near-term target is human-in-the-loop design, where optimization algorithms handle design space exploration while human designers guide objective formulation, constraint specification, and final selection among Pareto-optimal designs [221]. Longer-term, coupling computational optimization with digital twin feedback could enable soft robots that continuously refine their own design through operational experience.

## 7. Conclusions

This review has provided a broad, method-centric survey of bio-inspired optimization techniques for soft robot design, systematically covering evolutionary algorithms, swarm intelligence, topology optimization, and emerging machine-learning-assisted approaches. Our cross-method comparison and selection guidelines offer practical guidance for researchers to choose appropriate methods based on problem characteristics.

Several comparative findings emerge from this review. First, evolutionary algorithms remain the most widely adopted methods due to their flexibility in discrete, multi-modal design spaces, but their high evaluation cost makes simulation efficiency the primary bottleneck. Second, topology optimization produces high-resolution, fabrication-ready geometries for continuum structures but is largely restricted to gradient-available problems. Third, machine-learning-assisted approaches substantially reduce computational cost and enable simultaneous morphology-control co-optimization, yet most results lack experimental validation. Fourth, across all categories, only a small fraction of studies include fabrication and testing of optimized designs, confirming that the sim-to-real gap remains the field’s central open challenge.

Looking ahead, the convergence of surrogate-assisted optimization, GPU-accelerated simulation, fabrication-aware constraint integration, and standardized benchmarking is bringing the vision of autonomous design pipelines of soft robots closer to reality.

## Figures and Tables

**Table 1:** Comparison of the present review with existing reviews on soft robot design and optimization.

Review	Year	EA/SI	TO	ML	RL Co-Design	Generative Models	Cross-Method Comparison	Selection Guidelines	Tools & Benchmarks	Fabrication Integration
Rus & Tolley [5]	2015	∘	–	–	–	–	–	–	–	∘
Chen & Wang [35]	2020	✓	✓	–	–	–	∘	∘	∘	∘
Pinskier & Howard [36]	2022	✓	✓	–	∘	∘	–	–	∘	–
Ahmed et al. [37]	2022	∘	–	–	–	–	–	–	–	✓
Hammond et al. [38]	2023	∘	–	–	–	–	–	–	–	∘
Stroppa et al. [39]	2024	✓	∘	–	–	–	∘	∘	∘	–
Hasanshahi et al. [40]	2024	✓	✓	–	–	–	–	–	∘	–
Wang et al. [41]	2025	∘	–	–	–	–	–	–	–	∘
**Present review**	**2026**	✓	✓	✓	✓	✓	✓	✓	✓	✓

✓: covered in depth; ∘: partially covered; –: not covered. EA: evolutionary algorithms; SI: swarm intelligence; TO: topology optimization; ML: machine learning; RL: reinforcement learning.

**Table 2:** Comparison of bio-inspired optimization methods for soft robot design.

Method ^1^	Design Space Type	Typical Dimensionality	Gradient Requirement	FEM Evaluations Needed	Multi-Objective Support	Co-Design ^2^	References
GA	Continuous, discrete, mixed	Low–high	No	High	Strong	Possible	Pneumatic actuator geometry [92], gripper shape [93], voxel-based locomotion [21,162]
CMA-ES	Continuous	Low–mod.	No	Moderate	Limited	Limited	Fiber-reinforced actuators [99], chamber and wall thickness [41,100]
GP/CPPN-NEAT	Continuous, mixed	Low–high	No	High	Possible	Possible	Multi-material soft robots [21], reconfigurable organisms [25]
PSO	Continuous	Low–mod.	No	Moderate	Moderate	Limited	Bellows pneumatic actuator [109,111], controller tuning [111], sim-to-real calibration [112]
ACO	Discrete	Low–moderate	No	Mod.	Limited	No	Channel routing [113]
SA	Continuous, discrete	Low–moderate	No	Low–moderate	Weak	No	Compliant mechanism design [115]
DE	Continuous	Low–moderate	No	Moderate	Moderate	Limited	Compliant actuator [117], microgripper [118]
SIMP/Level-Set TO	Continuous (element densities)	Very high	Yes (adjoint)	Low	Possible	No	Soft gripper [53,122], PneuNet actuator [124], SoRoTop code [127]
BESO	Discrete (0/1)	Very high	Yes (sensitivity)	Low	Possible	No	Pneumatic actuator with pressure loads [137]
SAEO	Any (base EA)	Low–high	No (EA layer)	Low	Strong	Possible	Surrogate for pneumatic actuators [144], generalized surrogate pipeline [147]
BO	Continuous	Low	No	Very low	Moderate	Limited	Soft actuator tuning [150], multi-objective co-design [151]
RL Co-Design	Continuous, discrete	Moderate–high	Optional	High	Natural	Native	Evolution Gym [29], morphological pretraining [156], reconfigurable organisms [25]
Generative Models	Continuous (latent)	High	Yes (training)	High (training set)	Possible	Possible	DiffuseBot [26], modular robot GAN [161]

^1^ Qualitative assessments are relative comparisons across the listed methods, while specific evaluation ranges with supporting references are discussed in the accompanying text. ^2^ Co-Design refers to simultaneous morphology and control optimization.

**Table 3:** Taxonomy of bio-inspired optimization applications in soft robot design.

Application Domain	Optimization Methods	Biological Inspiration	Representative Work
Locomotion—crawling	GA/CPPN-NEAT, RL co-design, TO (MPM)	Caterpillar, worm, inchworm	[21,25,27,29,162,169]
Locomotion—swimming	GA, diff. simulation, shape opt.	Fish, jellyfish, manta ray	[9,170,171,172]
Locomotion—jumping	GA, CMA-ES	Frog, flea	[69,95]
Locomotion—walking	GA, TO, RL co-design, multi-obj. opt.	Quadruped, insect, turtle	[27,29,54,123,187]
Grippers—pneumatic	SIMP TO, GA, multi-material TO, MAP-Elites	Octopus arm, human hand	[122,174,175,176]
Grippers—motor-driven	SIMP TO, multi-obj. opt.	Biological grasping	[53,132,173,189]
Pneumatic actuators	SIMP TO, level-set, GA, BO, CNN surrogate	Muscular hydrostat	[14,92,124,125,141,190]
Compliant joints	SIMP TO, level-set, growth-inspired TO	Skeletal joints	[64,183,184]
Multi-material distribution	Multi-material TO, GA	Bone-tendon-muscle systems	[34,128,129,167,191]
Sensor-control co-design	Neural architecture search, RL	Biological proprioception	[84,168,187,188]

**Table 4:** Overview of simulation platforms used in bio-inspired optimization of soft robot design.

Platform	Physics Method	Diff.	Primary Use in Soft Robotics	Open Source	Typical Opt. Coupling
SOFA [30] (v23.06)	FEM (linear, corot.)	No	Real-time sim., control	Yes	EA, BO
FEniCS [32] (v0.8.0)	FEM (nonlinear)	Partial (adjoint)	High-fidelity TO	Yes	TO, EA
Sorotoki [166] (MATLAB) (v4.1)	FEM + Cosserat beam	Yes (adjoint)	Design, sim., TO, control	Yes	TO, EA, BO
ABAQUS (2026 FD01)/ANSYS/COMSOL [192]	FEM (nonlinear)	No	Validation, param. opt.	No	GA, PSO, CMA-ES
Voxelyze (v1.13) [31]/ Evosoro [162]	Mass-spring lattice	No	Voxel-based EA design	Yes	GA, CPPN-NEAT
voxcraft-sim [193]	Mass-spring lattice	No	Large-scale EA design	Yes	GA, CPPN-NEAT
Evolution Gym [29] (v1.0)	Mass-spring (2D)	Partial	Co-design benchmark	Yes	EA, RL
ChainQueen [83]	MPM	Yes	Diff. co-design	Yes	Gradient-based, RL
DiffTaichi [194] (v1.7)	MPM, FEM	Yes	Diff. co-design	Yes	Gradient-based, RL
MuJoCo [195] (v3.1)	Convex contact, FEM plugin	Partial (MJX)	RL control, co-design	Yes	RL
PyBullet [196] (v3.2)	Rigid + deformable body	No	RL control	Yes	RL
Isaac Gym [197] (Preview 4)	Rigid + FEM	Partial	Large-scale RL	Yes ^1^	RL
SoRoTop [127] (MATLAB)	FEM (2D, SIMP)	Yes (adjoint)	Pneumatic soft robot TO	Yes	TO (gradient-based)
TOPress/TOPress3D [126] (MATLAB)	FEM (2D/3D, SIMP)	Yes (adjoint)	Pressure-loaded TO	Yes	TO (gradient-based)

^1^ Isaac Gym is freely available but not fully open-source. Diff.: differentiable; Opt.: optimization; Param.: parameter; Corot.: corotational; Sim.: simulation. Platform characteristics were verified as of 1 September 2026, and may change with subsequent software releases.

## Data Availability

The data presented in this review paper encompasses an extensive analysis of the existing literature, including a critical review of materials. As this review involves synthesizing and interpreting information from published sources, the primary data consists of scholarly references, datasets, and analyses inherent in the reviewed literature. Researchers seeking further information are encouraged to inquire with the corresponding author at yilun_sun@tongji.edu.cn.

## Abbreviations

ACO	Ant Colony Optimization
BESO	Bi-directional Evolutionary Structural Optimization
BO	Bayesian Optimization
CMA-ES	Covariance Matrix Adaptation Evolution Strategy
CNN	Convolutional Neural Network
CPPN	Compositional Pattern-Producing Network
DE	Differential Evolution
DEA	Dielectric Elastomer Actuator
DIW	Direct Ink Writing
EA	Evolutionary Algorithm
FDM	Fused Deposition Modeling
FEM	Finite Element Method
GA	Genetic Algorithm
GP	Genetic Programming
MAP-Elites	Multi-dimensional Archive of Phenotypic Elites
ML	Machine Learning
MMA	Method of Moving Asymptotes
MOEA/D	Multi-Objective Evolutionary Algorithm based on Decomposition
MPM	Material Point Method
NEAT	Neuroevolution of Augmenting Topologies
NSGA-II	Non-dominated Sorting Genetic Algorithm II
OC	Optimality Criteria
PA	Polyamide
PSO	Particle Swarm Optimization
RL	Reinforcement Learning
SA	Simulated Annealing
SAEO	Surrogate-Assisted Evolutionary Optimization
SIMP	Solid Isotropic Material with Penalization
SLA	Stereolithography
SMP	Shape Memory Polymer
TO	Topology Optimization
TPE	Thermoplastic Elastomer
TPU	Thermoplastic Polyurethane
VAE	Variational Autoencoder

## References

[1] Kim S., Laschi C., Trimmer B. (2013). Soft robotics: A bioinspired evolution in robotics. Trends Biotechnol..

[2] Laschi C., Mazzolai B., Cianchetti M. (2016). Soft robotics: Technologies and systems pushing the boundaries of robot abilities. Sci. Robot..

[3] Kier W.M., Smith K.K. (1985). Tongues, tentacles and trunks: The biomechanics of movement in muscular-hydrostats. Zool. J. Linn. Soc..

[4] Trimmer B. (2014). A journal of soft robotics: Why now?. Soft Robot..

[5] Rus D., Tolley M.T. (2015). Design, fabrication and control of soft robots. Nature.

[6] Majidi C. (2014). Soft robotics: A perspective—Current trends and prospects for the future. Soft Robot..

[7] Polygerinos P., Correll N., Morin S.A., Mosadegh B., Onal C.D., Petersen K., Cianchetti M., Tolley M.T., Shepherd R.F. (2017). Soft robotics: Review of fluid-driven intrinsically soft devices; manufacturing, sensing, control, and applications in human-robot interaction. Adv. Eng. Mater..

[8] Yasa O., Toshimitsu Y., Michelis M.Y., Jones L.S., Filippi M., Buchner T., Katzschmann R.K. (2023). An overview of soft robotics. Annu. Rev. Control. Robot. Auton. Syst..

[9] Wang Y., Pancheri F., Lueth T.C., Sun Y. (2025). DuckyDog: An Erect Amphibious Robot with Variable-Stiffness Legs and Passive Fins. Adv. Intell. Syst..

[10] Webster R.J., Jones B.A. (2010). Design and kinematic modeling of constant curvature continuum robots: A review. Int. J. Robot. Res..

[11] Trivedi D., Rahn C.D., Kier W.M., Walker I.D. (2008). Soft robotics: Biological inspiration, state of the art, and future research. Appl. Bionics Biomech..

[12] Ogden R.W. (1997). Non-Linear Elastic Deformations.

[13] Marckmann G., Verron E. (2006). Comparison of hyperelastic models for rubber-like materials. Rubber Chem. Technol..

[14] Mosadegh B., Polygerinos P., Keplinger C., Wennstedt S., Shepherd R.F., Gupta U., Shim J., Bertoldi K., Walsh C.J., Whitesides G.M. (2014). Pneumatic networks for soft robotics that actuate rapidly. Adv. Funct. Mater..

[15] Hines L., Petersen K., Lum G.Z., Sitti M. (2017). Soft actuators for small-scale robotics. Adv. Mater..

[16] Gu G.Y., Zhu J., Zhu L.M., Zhu X. (2017). A survey on dielectric elastomer actuators for soft robots. Bioinspir. Biomim..

[17] Zhou X., Zhang Y., Qiu Z., Shan Z., Cai S., Bao G. (2024). Locomotion control of a rigid-soft coupled snake robot in multiple environments. Biomim. Intell. Robot..

[18] Duriez C. (2013). Control of elastic soft robots based on real-time finite element method. Proceedings of the 2013 IEEE International Conference on Robotics and Automation.

[19] Schegg P., Duriez C. (2022). Review on generic methods for mechanical modeling, simulation and control of soft robots. PLoS ONE.

[20] Hiller J., Lipson H. (2011). Automatic design and manufacture of soft robots. IEEE Trans. Robot..

[21] Cheney N., MacCurdy R., Clune J., Lipson H. (2014). Unshackling evolution: Evolving soft robots with multiple materials and a powerful generative encoding. ACM SIGEVOlution.

[22] Howard D., Eiben A.E., Kennedy D.F., Mouret J.B., Valencia P., Winkler D. (2019). Evolving embodied intelligence from materials to machines. Nat. Mach. Intell..

[23] Bäck T., Schwefel H.P. (1993). An overview of evolutionary algorithms for parameter optimization. Evol. Comput..

[24] Kennedy J., Eberhart R. (1995). Particle swarm optimization. Proceedings of the ICNN’95-International Conference on Neural Networks.

[25] Kriegman S., Blackiston D., Levin M., Bongard J. (2020). A scalable pipeline for designing reconfigurable organisms. Proc. Natl. Acad. Sci. USA.

[26] Wang T.H.J., Zheng J., Ma P., Du Y., Kim B., Spielberg A., Tenenbaum J., Gan C., Rus D. (2023). Diffusebot: Breeding soft robots with physics-augmented generative diffusion models. Adv. Neural Inf. Process. Syst..

[27] Kobayashi H., Gholami F., Montgomery S.M., Tanaka M., Yue L., Yuhn C., Sato Y., Kawamoto A., Qi H.J., Nomura T. (2024). Computational synthesis of locomotive soft robots by topology optimization. Sci. Adv..

[28] Matthews D., Spielberg A., Rus D., Kriegman S., Bongard J. (2023). Efficient automatic design of robots. Proc. Natl. Acad. Sci. USA.

[29] Bhatia J., Jackson H., Tian Y., Xu J., Matusik W. (2021). Evolution gym: A large-scale benchmark for evolving soft robots. Adv. Neural Inf. Process. Syst..

[30] Coevoet E., Morales-Bieze T., Largilliere F., Zhang Z., Thieffry M., Sanz-Lopez M., Carrez B., Marchal D., Goury O., Dequidt J. (2017). Software toolkit for modeling, simulation, and control of soft robots. Adv. Robot..

[31] Hiller J., Lipson H. (2014). Dynamic simulation of soft multimaterial 3d-printed objects. Soft Robot..

[32] Logg A., Mardal K.A., Wells G. (2012). Automated Solution of Differential Equations by the Finite Element Method: The FEniCS Book.

[33] Wallin T.J., Pikul J., Shepherd R.F. (2018). 3D printing of soft robotic systems: SOFT ROBOTICS. Nat. Rev. Mater..

[34] Schaffner M., Faber J.A., Pianegonda L., Rühs P.A., Coulter F., Studart A.R. (2018). 3D printing of robotic soft actuators with programmable bioinspired architectures. Nat. Commun..

[35] Chen F., Wang M.Y. (2020). Design optimization of soft robots: A review of the state of the art. IEEE Robot. Autom. Mag..

[36] Pinskier J., Howard D. (2022). From bioinspiration to computer generation: Developments in autonomous soft robot design. Adv. Intell. Syst..

[37] Ahmed F., Waqas M., Jawed B., Soomro A.M., Kumar S., Hina A., Khan U., Kim K.H., Choi K.H. (2022). Decade of bio-inspired soft robots: A review. Smart Mater. Struct..

[38] Hammond M., Cichella V., Lamuta C. (2023). Bioinspired soft robotics: State of the art, challenges, and future directions. Curr. Robot. Rep..

[39] Stroppa F., Majeed F.J., Batiya J., Baran E., Sarac M. (2024). Optimizing soft robot design and tracking with and without evolutionary computation: An intensive survey. Robotica.

[40] Hasanshahi B., Cao L., Song K.Y., Zhang W. (2024). Design of soft robots: A review of methods and future opportunities for research. Machines.

[41] Wang X., Wei R., Chen Z., Pang H., Li H., Yang Y., Hua Q., Shen G. (2025). Bioinspired intelligent soft robotics: From multidisciplinary integration to next-generation intelligence. Adv. Sci..

[42] Shepherd R.F., Ilievski F., Choi W., Morin S.A., Stokes A.A., Mazzeo A.D., Chen X., Wang M., Whitesides G.M. (2011). Multigait soft robot. Proc. Natl. Acad. Sci. USA.

[43] Pfeifer R., Lungarella M., Iida F. (2007). Self-organization, embodiment, and biologically inspired robotics. Science.

[44] Mengüç Y., Park Y.L., Pei H., Vogt D., Aubin P.M., Winchell E., Fluke L., Stirling L., Wood R.J., Walsh C.J. (2014). Wearable soft sensing suit for human gait measurement. Int. J. Robot. Res..

[45] Guan Q., Dai B., Sonar H., Hughes J. (2025). Inverse Co-Design of Mechanical and Sensory Properties in Soft Lattice Foams for Multifunctional Wearables. Adv. Sci..

[46] Case J.C., White E.L., Kramer R.K. (2015). Soft material characterization for robotic applications. Soft Robot..

[47] Yirmibesoglu O.D., Morrow J., Walker S., Gosrich W., Cañizares R., Kim H., Daalkhaijav U., Fleming C., Branyan C., Menguc Y. (2018). Direct 3D printing of silicone elastomer soft robots and their performance comparison with molded counterparts. Proceedings of the 2018 IEEE International Conference on Soft Robotics (RoboSoft).

[48] Tang J., Zhu Y., Jiang G., Xiao L., Ren W., Zhou Y., Gu Q., Yan B., Zhang J., Bi H. (2025). Human-centred design and fabrication of a wearable multimodal visual assistance system. Nat. Mach. Intell..

[49] Wen Z., Pancheri F., Wang Z., Lueth T.C., Sun Y. (2026). Enhancing Durability and Payload Capacity of Tendon-Driven Continuum Robots Using Multimaterial Soft-Rigid Hybrid Structure. IEEE/ASME Trans. Mechatron..

[50] Zhang H., Kumar A.S., Chen F., Fuh J.Y.H., Wang M.Y. (2019). Topology Optimized Multimaterial Soft Fingers for Applications on Grippers, Rehabilitation, and Artificial Hands. IEEE/ASME Trans. Mechatron..

[51] Liu C.H., Chung F.M., Chen Y., Chiu C.H., Chen T.L. (2020). Optimal Design of a Motor-Driven Three-Finger Soft Robotic Gripper. IEEE/ASME Trans. Mechatron..

[52] Sun Y., Wang Z., Lueth T.C. (2026). Optimization-based shape design of soft-rigid hybrid fingers for adaptive parallel robotic gripper. Biomim. Intell. Robot..

[53] Sun Y., Liu Y., Pancheri F., Lueth T.C. (2022). LARG: A Lightweight Robotic Gripper With 3-D Topology Optimized Adaptive Fingers. IEEE/ASME Trans. Mechatron..

[54] Sun Y., Pancheri F., Rehekampff C., Lueth T.C. (2024). TurBot: A turtle-inspired quadruped robot using topology optimized soft-rigid hybrid legs. IEEE/ASME Trans. Mechatron..

[55] Lee Y., Song W., Sun J.Y. (2020). Hydrogel soft robotics. Mater. Today Phys..

[56] López-Díaz A., Vázquez A.S., Vázquez E. (2024). Hydrogels in soft robotics: Past, present, and future. ACS Nano.

[57] Xia Y., He Y., Zhang F., Liu Y., Leng J. (2021). A review of shape memory polymers and composites: Mechanisms, materials, and applications. Adv. Mater..

[58] Zarek M., Layani M., Cooperstein I., Sachyani E., Cohn D., Magdassi S. (2015). 3D printing of shape memory polymers for flexible electronic devices. Adv. Mater..

[59] Ge Q., Sakhaei A.H., Lee H., Dunn C.K., Fang N.X., Dunn M.L. (2016). Multimaterial 4D printing with tailorable shape memory polymers. Sci. Rep..

[60] Pelrine R., Kornbluh R., Pei Q., Joseph J. (2000). High-speed electrically actuated elastomers with strain greater than 100%. Science.

[61] Chen F., Liu K., Wang Y., Zou J., Gu G., Zhu X. (2019). Automatic design of soft dielectric elastomer actuators with optimal spatial electric fields. IEEE Trans. Robot..

[62] Fang D., Ren F., Wang J., Li P., Cao L., Zhang J. (2024). A bionic robotic ankle driven by the multiple pneumatic muscle actuators. Biomim. Intell. Robot..

[63] Shi H., Tan K., Zhang B., Liu W. (2022). Review on research progress of hydraulic powered soft actuators. Energies.

[64] Sun Y., Liu Y., Lueth T.C. (2022). Optimization of Stress Distribution in Tendon-Driven Continuum Robots Using Fish-Tail-Inspired Method. IEEE Robot. Autom. Lett..

[65] In H., Cho K.J., Kim K., Lee B. (2011). Jointless structure and under-actuation mechanism for compact hand exoskeleton. Proceedings of the 2011 IEEE International Conference on Rehabilitation Robotics.

[66] Wang Y., Pancheri F., Lueth T.C., Sun Y. (2024). Design of a spider-inspired wheeled compliant leg for search mobile robots. Biomim. Intell. Robot..

[67] Shahinpoor M., Kim K.J. (2001). Ionic polymer-metal composites: I. Fundamentals. Smart Mater. Struct..

[68] Yu J., Wang J., Li S., Wang X., Liao X., Shuai P., Wu Z. (2026). Robotic fish with tunable stiffness soft tail based on bionic spinal muscle system. Biomim. Intell. Robot..

[69] Tolley M.T., Shepherd R.F., Karpelson M., Bartlett N.W., Galloway K.C., Wehner M., Nunes R., Whitesides G.M., Wood R.J. (2014). An untethered jumping soft robot. Proceedings of the 2014 IEEE/RSJ International Conference on Intelligent Robots and Systems.

[70] Kim Y., Yuk H., Zhao R., Chester S.A., Zhao X. (2018). Printing ferromagnetic domains for untethered fast-transforming soft materials. Nature.

[71] Schmitt F., Piccin O., Barbé L., Bayle B. (2018). Soft robots manufacturing: A review. Front. Robot. AI.

[72] Pancheri F., Lueth T.C., Sun Y. (2026). Enabling Metal-Based Soft Robotics Through Investment Casting. Adv. Intell. Syst..

[73] Wang D., Wang J., Shen Z., Jiang C., Zou J., Dong L., Fang N.X., Gu G. (2023). Soft actuators and robots enabled by additive manufacturing. Annu. Rev. Control. Robot. Auton. Syst..

[74] Liu C., Cui B., Huang X., Zheng X., Vo-Doan T.T., Qiao R. (2025). 3D Printing of Soft Robotic Systems: Advances in Fabrication Strategies and Future Trends. Adv. Robot. Res..

[75] Raj R., Song Q.C., Juang J.Y. (2026). Multi-material additive manufacturing of soft robotic systems: A comprehensive review. Adv. Robot. Res..

[76] Liu H., Wu C., Lin S., Lam J., Xi N., Chen Y. (2025). Advances in 3D and 4D printing of soft robotics and their applications. Adv. Intell. Syst..

[77] Zhu K., Lueth T.C., Sun Y. (2025). Design of FDM-printable tendon-driven continuum robots using a serial S-shaped backbone structure. Biomim. Intell. Robot..

[78] Fu J., Wang Z., Pancheri F., Lueth T.C., Sun Y. (2026). Optimization-based automated generation of 1-DOF multi-section continuum robots with predefined end-effector poses. Biomim. Intell. Robot..

[79] Whitney J.P., Sreetharan P.S., Ma K.Y., Wood R.J. (2011). Pop-up book MEMS. J. Micromech. Microeng..

[80] Gafford J., Ding Y., Harris A., McKenna T., Polygerinos P., Holland D., Walsh C., Moser A. (2015). Shape deposition manufacturing of a soft, atraumatic, and deployable surgical grasper. J. Mech. Robot..

[81] Qin L., Peng H., Huang X., Liu M., Huang W. (2024). Modeling and simulation of dynamics in soft robotics: A review of numerical approaches. Curr. Robot. Rep..

[82] Nocedal J., Wright S.J. (2006). Numerical Optimization.

[83] Hu Y., Liu J., Spielberg A., Tenenbaum J.B., Freeman W.T., Wu J., Rus D., Matusik W. (2019). Chainqueen: A real-time differentiable physical simulator for soft robotics. Proceedings of the 2019 International Conference on Robotics and Automation (ICRA).

[84] Spielberg A., Amini A., Chin L., Matusik W., Rus D. (2021). Co-learning of task and sensor placement for soft robotics. IEEE Robot. Autom. Lett..

[85] Deb K., Pratap A., Agarwal S., Meyarivan T. (2002). A fast and elitist multiobjective genetic algorithm: NSGA-II. IEEE Trans. Evol. Comput..

[86] Liu J., Li M., Wang Y., Zhao D., Deng R. (2024). Multi-gait snake robot for inspecting inner wall of a pipeline. Biomim. Intell. Robot..

[87] Huang H., Xian S., Xiong C., Li W., Zhong Y. (2025). Design and dynamics modeling of a hybrid drive bionic robotic fish. Biomim. Intell. Robot..

[88] Eiben A.E., Smith J.E. (2003). Introduction to Evolutionary Computing.

[89] Slowik A., Kwasnicka H. (2020). Evolutionary algorithms and their applications to engineering problems. Neural Comput. Appl..

[90] Lambora A., Gupta K., Chopra K. (2019). Genetic algorithm-A literature review. Proceedings of the 2019 International Conference on Machine Learning, Big Data, Cloud and Parallel Computing (COMITCon).

[91] Alhijawi B., Awajan A. (2024). Genetic algorithms: Theory, genetic operators, solutions, and applications. Evol. Intell..

[92] Runge G., Peters J., Raatz A. (2017). Design optimization of soft pneumatic actuators using genetic algorithms. Proceedings of the 2017 IEEE International Conference on Robotics and Biomimetics (ROBIO).

[93] Datta R., Pradhan S., Bhattacharya B. (2015). Analysis and design optimization of a robotic gripper using multiobjective genetic algorithm. IEEE Trans. Syst. Man Cybern. Syst..

[94] Mosser L., Barbé L., Rubbert L., Renaud P. (2023). Towards automatic design of soft pneumatic actuators: Inner structure design using cnn model and bézier curve-based genetic algorithm. IEEE Robot. Autom. Lett..

[95] Cheney N., Clune J., Lipson H. Evolved electrophysiological soft robots. Proceedings of the Artificial Life Conference Proceedings.

[96] Omarov B.S., Ibrayev S., Ibrayeva A., Amanov B., Momynkulov Z. (2024). Optimal Leg Linkage Design for Horizontal Propel of a Walking Robot Using Non-Dominated Sorting Genetic Algorithm. IEEE Access.

[97] Hansen N., Ostermeier A. (1996). Adapting arbitrary normal mutation distributions in evolution strategies: The covariance matrix adaptation. Proceedings of the IEEE International Conference On Evolutionary Computation.

[98] Hansen N., Müller S.D., Koumoutsakos P. (2003). Reducing the time complexity of the derandomized evolution strategy with covariance matrix adaptation (CMA-ES). Evol. Comput..

[99] Connolly F., Walsh C.J., Bertoldi K. (2017). Automatic design of fiber-reinforced soft actuators for trajectory matching. Proc. Natl. Acad. Sci. USA.

[100] Morzadec T., Marcha D., Duriez C. (2019). Toward shape optimization of soft robots. Proceedings of the 2019 2nd IEEE International Conference on Soft Robotics (RoboSoft).

[101] Espejo P.G., Ventura S., Herrera F. (2009). A survey on the application of genetic programming to classification. IEEE Trans. Syst. Man. Cybern. C Appl. Rev..

[102] Stanley K.O. (2007). Compositional pattern producing networks: A novel abstraction of development. Genet. Program. Evolvable Mach..

[103] Ellis D.R., Venter M.P., Venter G. (2022). Generative design procedure for embedding specified planar behavior in modular soft pneumatic actuators. Soft Robot..

[104] Venter M.P., Conradie N.T. (2023). Intermediate Encoding Layers for the Generative Design of 2D Soft Robot Actuators: A Comparison of CPPN’s, L-Systems and Random Generation. Math. Comput. Appl..

[105] Stanley K.O., Miikkulainen R. (2002). Evolving neural networks through augmenting topologies. Evol. Comput..

[106] Bonabeau E., Dorigo M., Theraulaz G. (1999). Swarm Intelligence: From Natural to Artificial Systems.

[107] Chakraborty A., Kar A.K. (2017). Swarm intelligence: A review of algorithms. Nature-Inspired Computing and Optimization.

[108] Tang J., Liu G., Pan Q. (2021). A review on representative swarm intelligence algorithms for solving optimization problems: Applications and trends. IEEE/CAA J. Autom. Sin..

[109] Liang L., Meng L., Ma H. (2023). Design, Characterization, and Optimization of the Soft Bellows Pneumatic Actuator. Proceedings of the 2023 IEEE International Conference on Mechatronics and Automation (ICMA).

[110] Bodily D.M., Allen T.F., Killpack M.D. (2017). Multi-objective design optimization of a soft, pneumatic robot. Proceedings of the 2017 IEEE International Conference on Robotics and Automation (ICRA).

[111] Meng H., Zhang S., Zhang W., Ren Y. (2024). Optimizing actual PID control for walking quadruped soft robots using genetic algorithms. Sci. Rep..

[112] Sant’Anna T., Matos V.S., da Silva L.C. Optimization of soft manipulator simulation using a particle swarm approach. Proceedings of the Congresso Brasileiro de Automática-CBA.

[113] Dorigo M., Maniezzo V., Colorni A. (1996). Ant system: Optimization by a colony of cooperating agents. IEEE Trans. Syst. Man Cybern. B Cybern..

[114] Kirkpatrick S., Gelatt C.D., Vecchi M.P. (1983). Optimization by simulated annealing. Science.

[115] Saxena A., Ananthasuresh G. (2000). On an optimal property of compliant topologies. Struct. Multidiscip. Optim..

[116] Storn R., Price K. (1997). Differential evolution–a simple and efficient heuristic for global optimization over continuous spaces. J. Glob. Optim..

[117] Torrealba R.R., Udelman S.B. (2016). Design of cam shape for maximum stiffness variability on a novel compliant actuator using differential evolution. Mech. Mach. Theory.

[118] Dao T.P., Huang S.C., Le Chau N. (2018). Robust parameter design for a compliant microgripper based on hybrid Taguchi-differential evolution algorithm. Microsyst. Technol..

[119] Bendsøe M.P., Sigmund O. (2004). Topology Optimization: Theory, Methods, and Applications.

[120] Sigmund O., Maute K. (2013). Topology optimization approaches: A comparative review. Struct. Multidiscip. Optim..

[121] Sigmund O. (2001). A 99 line topology optimization code written in Matlab. Struct. Multidiscip. Optim..

[122] Zhang H., Wang M.Y., Chen F., Wang Y., Kumar A.S., Fuh J.Y. (2017). Design and development of a soft gripper with topology optimization. Proceedings of the 2017 IEEE/RSJ International Conference On Intelligent Robots and Systems (IROS).

[123] Sun Y., Zong C., Pancheri F., Chen T., Lueth T.C. (2023). Design of topology optimized compliant legs for bio-inspired quadruped robots. Sci. Rep..

[124] Caasenbrood B., Pogromsky A., Nijmeijer H. A Computational Design Framework for Pressure-driven Soft Robots through Nonlinear Topology Optimization. Proceedings of the 2020 3rd IEEE International Conference on Soft Robotics (RoboSoft).

[125] Kumar P., Frouws J.S., Langelaar M. (2020). Topology optimization of fluidic pressure-loaded structures and compliant mechanisms using the Darcy method. Struct. Multidiscip. Optim..

[126] Kumar P. (2023). TOPress: A MATLAB implementation for topology optimization of structures subjected to design-dependent pressure loads. Struct. Multidiscip. Optim..

[127] Kumar P. (2024). SoRoTop: A hitchhiker’s guide to topology optimization MATLAB code for design-dependent pneumatic-driven soft robots. Optim. Eng..

[128] Zhang H., Kumar A.S., Fuh J.Y., Wang M.Y. (2018). Topology optimized design, fabrication and evaluation of a multimaterial soft gripper. Proceedings of the 2018 IEEE International Conference on Soft Robotics (RoboSoft).

[129] Kumar P., Pinskier J., Howard D., Langelaar M. (2023). Topology optimization of fluidic pressure-driven multi-material compliant mechanisms. Proceedings of the International Design Engineering Technical Conferences and Computers and Information in Engineering Conference.

[130] Wang M.Y., Wang X., Guo D. (2003). A level set method for structural topology optimization. Comput. Methods Appl. Mech. Eng..

[131] Van Dijk N.P., Maute K., Langelaar M., Van Keulen F. (2013). Level-set methods for structural topology optimization: A review. Struct. Multidiscip. Optim..

[132] Chen F., Xu W., Zhang H., Wang Y., Cao J., Wang M.Y., Ren H., Zhu J., Zhang Y. (2018). Topology optimized design, fabrication, and characterization of a soft cable-driven gripper. IEEE Robot. Autom. Lett..

[133] Chen S., Chen F., Cao Z., Wang Y., Miao Y., Gu G., Zhu X. (2021). Topology optimization of skeleton-reinforced soft pneumatic actuators for desired motions. IEEE/ASME Trans. Mechatron..

[134] Picelli R., Neofytou A., Kim H.A. (2019). Topology optimization for design-dependent hydrostatic pressure loading via the level-set method. Struct. Multidiscip. Optim..

[135] Huang X., Xie Y.M. (2009). Bi-directional evolutionary topology optimization of continuum structures with one or multiple materials. Comput. Mech..

[136] Huang X., Xie M. (2010). Evolutionary Topology Optimization of Continuum Structures: Methods and Applications.

[137] Chen Y., Xia Z., Zhao Q. (2019). Optimal design of soft pneumatic bending actuators subjected to design-dependent pressure loads. IEEE/ASME Trans. Mechatron..

[138] Mattheck C. (1998). Design in Nature: Learning From Trees.

[139] Bocko J., Delyová I., Kostka J., Sivák P., Fil’o M. (2024). Shape optimization of structures by biological growth method. Appl. Sci..

[140] Sun Y., Xu L., Yang J., Lueth T.C. (2019). Automatic design in matlab using pde toolbox for shape and topology optimization. Proceedings of the ASME International Mechanical Engineering Congress and Exposition.

[141] Dalklint A., Wallin M., Tortorelli D. (2024). Simultaneous shape and topology optimization of inflatable soft robots. Comput. Methods Appl. Mech. Eng..

[142] Jin Y. (2011). Surrogate-assisted evolutionary computation: Recent advances and future challenges. Swarm Evol. Comput..

[143] Forrester A.I., Keane A.J. (2009). Recent advances in surrogate-based optimization. Prog. Aerosp. Sci..

[144] Beaber S.I., Liu Z., Sun Y. (2024). Physics-guided deep learning enabled surrogate modeling for pneumatic soft robots. IEEE Robot. Autom. Lett..

[145] Zhang C., Sedal A., Zhao Y.F. (2023). Differentiable surrogate models for design and trajectory optimization of auxetic soft robots. Proceedings of the 2023 IEEE International Conference on Soft Robotics (RoboSoft).

[146] Pu Y., Hu X., Liu X., Niu S., An N. (2026). Digital twins for soft pneumatic actuators via reduced-order surrogate modeling. Int. J. Solids Struct..

[147] Yao Y., Howard D., Maiolino P. (2026). Generalized Task-Driven Design of Soft Robots via Reduced-Order Finite Element Method-Based Surrogate Modeling. Adv. Intell. Syst..

[148] Shahriari B., Swersky K., Wang Z., Adams R.P., De Freitas N. (2015). Taking the human out of the loop: A review of Bayesian optimization. Proc. IEEE.

[149] Candelieri A. (2021). A gentle introduction to bayesian optimization. Proceedings of the 2021 Winter Simulation Conference (WSC).

[150] Yao Y., Chen Y., He L., Maiolino P. (2023). Design optimization for bellow soft pneumatic actuators in shape-matching. Proceedings of the 2023 IEEE International Conference on Soft Robotics (RoboSoft).

[151] Wang X., Wang B., Pinskier J., Xie Y., Brett J., Scalzo R., Howard D. (2024). Fin-bayes: A multi-objective bayesian optimization framework for soft robotic fingers. Soft Robot..

[152] Kim Y., Pan Z., Hauser K. (2021). Mo-bbo: Multi-objective bilevel bayesian optimization for robot and behavior co-design. Proceedings of the 2021 IEEE International Conference on Robotics and Automation (ICRA).

[153] Ha D. (2019). Reinforcement learning for improving agent design. Artif. Life.

[154] Schaff C., Yunis D., Chakrabarti A., Walter M.R. (2019). Jointly learning to construct and control agents using deep reinforcement learning. Proceedings of the 2019 International Conference on Robotics and Automation (ICRA).

[155] Bächer M., Knoop E., Schumacher C. (2021). Design and control of soft robots using differentiable simulation. Curr. Robot. Rep..

[156] Strgar L., Kriegman S. Accelerated co-design of robots through morphological pretraining. Proceedings of the International Conference on Learning Representations.

[157] Wang J., Jiang M., Ramirez L.A., Yang B., Zhang M., Figueroa E., Yan W., Kramer-Bottiglio R. (2025). Surrogate compliance modeling enables reinforcement learned locomotion gaits for soft robots. arXiv.

[158] Goodfellow I., Pouget-Abadie J., Mirza M., Xu B., Warde-Farley D., Ozair S., Courville A., Bengio Y. (2020). Generative adversarial networks. Commun. ACM.

[159] Kingma D.P., Welling M. (2013). Auto-encoding variational bayes. arXiv.

[160] Christiansen M.B., Rafsanjani A., Jørgensen J. (2024). Nature redux: Interrogating biomorphism and soft robot aesthetics through generative AI. Front. Robot. AI.

[161] Hu J., Whitman J., Travers M., Choset H. (2022). Modular robot design optimization with generative adversarial networks. Proceedings of the 2022 International Conference on Robotics and Automation (ICRA).

[162] Medvet E., Bartoli A., Pigozzi F., Rochelli M. Biodiversity in evolved voxel-based soft robots. Proceedings of the Genetic and Evolutionary Computation Conference.

[163] Wang F., Lazarov B.S., Sigmund O. (2011). On projection methods, convergence and robust formulations in topology optimization. Struct. Multidiscip. Optim..

[164] Liu J., Ma Y. (2016). A survey of manufacturing oriented topology optimization methods. Adv. Eng. Softw..

[165] Sun Y., Liu Y., Zhou N., Lueth T.C. A MATLAB-Based Framework for Designing 3D Topology Optimized Soft Robotic Grippers. Proceedings of the 2021 IEEE/ASME International Conference on Advanced Intelligent Mechatronics (AIM).

[166] Caasenbrood B.J., Pogromsky A.Y., Nijmeijer H. (2024). Sorotoki: A Matlab toolkit for design, modeling, and control of soft robots. IEEE Access.

[167] Guan Q., Dai B., Cheng H.H., Hughes J. (2025). Lattice structure musculoskeletal robots: Harnessing programmable geometric topology and anisotropy. Sci. Adv..

[168] Ochieze C., Liu Z., Sun Y. (2025). Distributed Sensing Enabled Embodied Intelligence for Soft Finger Manipulation. Actuators.

[169] Liu X., Song M., Fang Y., Zhao Y., Cao C. (2022). Worm-inspired soft robots enable adaptable pipeline and tunnel inspection. Adv. Intell. Syst..

[170] Corucci F., Cheney N., Giorgio-Serchi F., Bongard J., Laschi C. (2018). Evolving soft locomotion in aquatic and terrestrial environments: Effects of material properties and environmental transitions. Soft Robot..

[171] Ma P., Du T., Zhang J.Z., Wu K., Spielberg A., Katzschmann R.K., Matusik W. (2021). Diffaqua: A differentiable computational design pipeline for soft underwater swimmers with shape interpolation. ACM Trans. Graph. (TOG).

[172] Laschi C., Cianchetti M., Mazzolai B., Margheri L., Follador M., Dario P. (2012). Soft robot arm inspired by the octopus. Adv. Robot..

[173] Liu C.H., Chen T.L., Chiu C.H., Hsu M.C., Chen Y., Pai T.Y., Peng W.G., Chiang Y.P. (2018). Optimal design of a soft robotic gripper for grasping unknown objects. Soft Robot..

[174] Pinskier J., Kumar P., Langelaar M., Howard D. (2023). Automated design of pneumatic soft grippers through design-dependent multi-material topology optimization. Proceedings of the 2023 IEEE International Conference on Soft Robotics (RoboSoft).

[175] Pinskier J., Wang X., Liow L., Xie Y., Kumar P., Langelaar M., Howard D. (2024). Diversity-based topology optimization of soft robotic grippers. Adv. Intell. Syst..

[176] Xie Y., Pinskier J., Liow L., Howard D., Iida F. (2024). A’MAP’to find high-performing soft robot designs: Traversing complex design spaces using MAP-elites and Topology Optimization. Proceedings of the 2024 IEEE/RSJ International Conference on Intelligent Robots and Systems (IROS).

[177] Zhang X., Zhu B. (2018). Topology Optimization of Compliant Mechanisms.

[178] Megaro V., Zehnder J., Bächer M., Coros S., Gross M.H., Thomaszewski B. (2017). A computational design tool for compliant mechanisms. ACM Trans. Graph..

[179] Sun Y., Lueth T.C. (2023). Design of 3d-printed continuum robots using topology optimized compliant joints. Proceedings of the 2023 IEEE International Conference on Soft Robotics (RoboSoft).

[180] Sigmund O. (1997). On the design of compliant mechanisms using topology optimization. J. Struct. Mech..

[181] Mutlu R., Alici G., in het Panhuis M., Spinks G.M. (2016). 3D printed flexure hinges for soft monolithic prosthetic fingers. Soft Robot..

[182] Hussain I., Iqbal Z., Malvezzi M., Prattichizzo D., Salvietti G. (2019). How to 3d-print compliant joints with a selected stiffness for cooperative underactuated soft grippers. Proceedings of the International Workshop on Human-Friendly Robotics.

[183] Sun Y., Lueth T.C. (2023). Enhancing torsional stiffness of continuum robots using 3-D topology optimized flexure joints. IEEE/ASME Trans. Mechatron..

[184] Chen S., Zhou G., Li D., Song Z., Chen F. (2023). Soft robotic joints with anisotropic stiffness by multiobjective topology optimization. IEEE/ASME Trans. Mechatron..

[185] Kuang X., Wu J., Chen K., Zhao Z., Ding Z., Hu F., Fang D., Qi H.J. (2019). Grayscale digital light processing 3D printing for highly functionally graded materials. Sci. Adv..

[186] Clausen A., Wang F., Jensen J.S., Sigmund O., Lewis J.A. (2015). Topology optimized architectures with programmable Poisson’s ratio over large deformations. Adv. Mater..

[187] Wang J., Pan S., Serra-Gomez A., Wei X., Xie Y. (2026). Evolving Embodied Intelligence: Graph Neural Network–Driven Co-Design of Morphology and Control in Soft Robotics. arXiv.

[188] Truby R.L., Della Santina C., Rus D. (2020). Distributed proprioception of 3D configuration in soft, sensorized robots via deep learning. IEEE Robot. Autom. Lett..

[189] Wang R., Zhang X., Zhu B., Zhang H., Chen B., Wang H. (2020). Topology optimization of a cable-driven soft robotic gripper. Struct. Multidiscip. Optim..

[190] Chi J.C., Liu C.H. (2025). Topology optimization of a soft pneumatic actuator considering design-dependent loads. IEEE/ASME Trans. Mechatron..

[191] Chen F., Chen S., Wang Y., Li D., Song Z., Gu G., Zhu X. (2024). Multimaterial soft gripper design with dual-mode pinches for grasping in confined spaces. IEEE/ASME Trans. Mechatron..

[192] Kusay D., Aydın M., COŞKUN Y. (2024). Topology optimization for flexible robotic gripper using ANSYS. J. Mater. Electron. Devices.

[193] Liu S., Matthews D., Kriegman S., Bongard J. (2020). Voxcraft-Sim, a GPU-Accelerated Voxel-Based Physics Engine. https://github.com/voxcraft/voxcraft-sim.

[194] Hu Y., Anderson L., Li T.M., Sun Q., Carr N., Ragan-Kelley J., Durand F. (2019). Difftaichi: Differentiable programming for physical simulation. arXiv.

[195] Todorov E., Erez T., Tassa Y. (2012). Mujoco: A physics engine for model-based control. Proceedings of the 2012 IEEE/RSJ International Conference On Intelligent Robots and Systems.

[196] Coumans E., Bai Y. PyBullet, a Python Module for Physics Simulation for Games, Robotics and Machine Learning. 2016–2021. https://pypi.org/project/pybullet/.

[197] Makoviychuk V., Wawrzyniak L., Guo Y., Lu M., Storey K., Macklin M., Hoeller D., Rudin N., Allshire A., Handa A. (2021). Isaac gym: High performance gpu-based physics simulation for robot learning. arXiv.

[198] Duriez C., Coevoet E., Largilliere F., Morales-Bieze T., Zhang Z., Sanz-Lopez M., Carrez B., Marchal D., Goury O., Dequidt J. (2016). Framework for online simulation of soft robots with optimization-based inverse model. Proceedings of the 2016 IEEE International Conference On Simulation, Modeling, and Programming for Autonomous Robots (SIMPAR).

[199] Ferrentino P., Lopez-Diaz A., Terryn S., Legrand J., Brancart J., Van Assche G., Vazquez E., Vazquez A., Vanderborght B. (2022). Quasi-static FEA model for a multi-material soft pneumatic actuator in SOFA. IEEE Robot. Autom. Lett..

[200] Ferrentino P., Roels E., Brancart J., Terryn S., Van Assche G., Vanderborght B. (2023). Finite element analysis-based soft robotic modeling: Simulating a soft actuator in sofa. IEEE Robot. Autom. Mag..

[201] Wehner C., Schubert F., Hellkamp H., Hahnewald J., Schaefer K., Khan M.B., Gutfleisch O. (2025). An Open-Source User-Friendly Interface for Simulating Magnetic Soft Robots using Simulation Open Framework Architecture (SOFA). arXiv.

[202] Alnæs M., Blechta J., Hake J., Johansson A., Kehlet B., Logg A., Richardson C., Ring J., Rognes M.E., Wells G.N. (2015). The FEniCS project version 1.5. Arch. Numer. Softw..

[203] Moss A., Sholl N., Mohseni K. (2026). Utilizing Spatially Varying Fiber Arrays in Soft Morphing Surfaces for Grasping Applications. J. Mech. Robot..

[204] de Souza E.M., Silva E.C.N. (2020). Topology optimization applied to the design of actuators driven by pressure loads. Struct. Multidiscip. Optim..

[205] Chen Q., Zhang X., Zhu B. (2019). A 213-line topology optimization code for geometrically nonlinear structures. Struct. Multidiscip. Optim..

[206] Kriegman S., Cappelle C., Corucci F., Bernatskiy A., Cheney N., Bongard J.C. Simulating the evolution of soft and rigid-body robots. Proceedings of the Genetic and Evolutionary Computation Conference Companion.

[207] Liu S., Yao W., Wang H., Peng W., Yang Y. (2023). Rapidly evolving soft robots via action inheritance. IEEE Trans. Evol. Comput..

[208] Xie Y., Yan X., Iida F. Accelerating soft robot evolution using n-gram-based controller inheritance and genetic co-design. Proceedings of the Genetic and Evolutionary Computation Conference Companion.

[209] Xu J., Chen T., Zlokapa L., Foshey M., Matusik W., Sueda S., Agrawal P. An End-to-End Differentiable Framework for Contact-Aware Robot Design. Proceedings of the Robotics: Science and Systems 2021.

[210] Talischi C., Paulino G.H., Pereira A., Menezes I.F. (2012). PolyTop: A Matlab implementation of a general topology optimization framework using unstructured polygonal finite element meshes. Struct. Multidiscip. Optim..

[211] Liu K., Tovar A. (2014). An efficient 3D topology optimization code written in Matlab. Struct. Multidiscip. Optim..

[212] Wang C., Zhao Z., Zhou M., Sigmund O., Zhang X.S. (2021). A comprehensive review of educational articles on structural and multidisciplinary optimization. Struct. Multidiscip. Optim..

[213] Wang Y., Li X., Long K., Wei P. (2023). Open-source codes of topology optimization: A summary for beginners to start their research. Comput. Model. Eng. Sci..

[214] Tavakoli R., Mohseni S.M. (2014). Alternating active-phase algorithm for multimaterial topology optimization problems: A 115-line MATLAB implementation. Struct. Multidiscip. Optim..

[215] Zheng R., Yi B., Peng X., Yoon G.H. (2024). An efficient code for the multi-material topology optimization of 2D/3D continuum structures written in MATLAB. Appl. Sci..

[216] Aage N., Nobel-Jørgensen M., Andreasen C.S., Sigmund O. (2013). Interactive topology optimization on hand-held devices. Struct. Multidiscip. Optim..

[217] Sun Y., Zhang D., Liu Y., Lueth T.C. (2020). FEM-Based Mechanics Modeling of Bio-Inspired Compliant Mechanisms for Medical Applications. IEEE Trans. Med. Robot. Bionics.

[218] Diani J., Fayolle B., Gilormini P. (2009). A review on the Mullins effect. Eur. Polym. J..

[219] Tao F., Cheng J., Qi Q., Zhang M., Zhang H., Sui F. (2018). Digital twin-driven product design, manufacturing and service with big data. Int. J. Adv. Manuf. Technol..

[220] Zhang Q., Li H. (2007). MOEA/D: A multiobjective evolutionary algorithm based on decomposition. IEEE Trans. Evol. Comput..

[221] Takagi H. (2001). Interactive evolutionary computation: Fusion of the capabilities of EC optimization and human evaluation. Proc. IEEE.

